# Preserved α2 oscillations underpin reserve-related resilience of semantic verbal fluency in multiple sclerosis

**DOI:** 10.3389/fnins.2026.1869738

**Published:** 2026-07-22

**Authors:** Alexandra Anagnostopoulou, Panagiotis E. Kartsidis, Nefeli Tsoukaki, Maria Karagianni, Nikos Melanitis, Athanasia Liozidou, Ioannis Nikolaidis, Christos Chatzichristos, Vahe Poghosyan, Nikolaos Grigoriadis, Leontios J. Hadjileontiadis, Panagiotis D. Bamidis, Charis Styliadis

**Affiliations:** 1 Medical Physics and Digital Innovation Laboratory, Aristotle University of Thessaloniki, Thessaloniki, Greece; 2 Department of Electrical and Computer Engineering, Aristotle University of Thessaloniki, Thessaloniki, Greece; 3 AINIGMA Technologies, Innovation and Incubation Center KU Leuven, Leuven, Belgium; 4 Laboratory of Cognitive Neuroscience and Clinical Neuropsychology, Scientific College of Greece, Athens, Greece; 5 1st Neurology Department, Athens Medical School, National and Kapodistrian University of Athens, Athens, Greece; 6 Department of Neurology, Hippokration General Hospital of Thessaloniki, Thessaloniki, Greece; 7 Department of Electrical Engineering, STADIUS Center for Dynamical Systems, Signal Processing and Data Analytics, KU Leuven, Leuven, Belgium; 8 Department of Neurophysiology, National Neuroscience Institute, King Fahad Medical City, Riyadh, Saudi Arabia; 9 Multiple Sclerosis Center, 2nd Department of Neurology, Aristotle University of Thessaloniki, Thessaloniki, Greece; 10 Department of Biomedical Engineering and Biotechnology, Khalifa University of Science and Technology, Abu Dhabi, United Arab Emirates

**Keywords:** cognitive reserve, EEG, mediation/moderation, multiple sclerosis, semantic verbal fluency, α2 oscillations

## Abstract

**Introduction:**

Cognitive reserve (CR) is associated with better cognitive outcomes in people with multiple sclerosis (PwMS), yet the neurophysiological factors mediating this relationship remain poorly understood. In this context, alpha (*α*) oscillatory activity has been linked to both CR and semantic processing, making it a plausible neural mediator of reserve-related resilience. We investigated whether resting-state α activity statistically mediates the relationship between CR and semantic verbal fluency (SVF) in PwMS.

**Methods:**

Sixty-four PwMS and 46 age- and sex-matched healthy controls underwent resting-state electroencephalography to compute cortical global spectral power. CR was assessed using the Cognitive Reserve Index questionnaire, and verbal fluency was evaluated with the semantic and phonemic verbal fluency tests. Regression and mediation analyses examined associations among CR, spectral power, and verbal fluency, adjusted for demographic, clinical, and patient-reported factors. Exploratory moderated mediation analyses assessed potential variation across levels of disability and perceived disease impact.

**Results:**

PwMS exhibited reduced upper α power (α2; 10–13 Hz) compared with healthy controls. α2 power statistically mediated the relationship between CR and SVF, such that individuals with higher CR showed α2 activity closer to healthy norms, which in turn was associated with better SVF performance. No such associations were observed for phonemic verbal fluency, and this mediation was not replicated in healthy controls. Regional analyses indicated that these indirect effects were most evident in bilateral frontal, left central, and right parietal regions. The mediation was not formally moderated by disease burden, although conditional indirect effects suggested the CR → α2 → SVF path was present at moderate and moderate-to-high levels of physical disability and perceived disease impact, respectively.

**Discussion:**

These findings suggest that preserved α2 oscillatory activity is a candidate neurophysiological mechanism linking CR and semantic-language functioning in PwMS. To the best of our knowledge, this is the first study in MS to jointly examine CR, oscillatory preservation, and SVF within a single analytical framework, advancing current understanding of the neural basis of reserve-related resilience in PwMS.

**Clinical trial registration:**

ClinicalTrials.gov, identifier NCT04806568.

## Introduction

Cognitive decline affects approximately two-thirds of people with multiple sclerosis (PwMS) and represents a major determinant of functional independence, employment, and quality of life (Benedict et al., 2020). Cognitive outcomes differ substantially across PwMS even when structural disease burden (e.g., lesion load, gray matter atrophy) is comparable (Eijlers et al., 2018), suggesting that factors beyond the pathological substrate contribute to this inter-individual variation. One framework proposed to explain this variability in MS is cognitive reserve (CR) (Sumowski and Leavitt, 2013).

CR reflects the cumulative influence of lifelong experiences, such as education, occupational complexity, and engagement in intellectually stimulating leisure activities, which confer resilience against the cognitive effects of brain damage or neurodegeneration (Stern, 2012). In PwMS, higher CR offers protection against cognitive impairment (Amato et al., 2019; Santangelo et al., 2019a for a meta-analysis) and the adverse effects of structural damage (Benedict et al., 2010; Modica et al., 2016; Santangelo et al., 2019b; Sumowski and Leavitt, 2013), with particularly strong benefits in cognitive functions relying on long-term memory systems, including verbal fluency (VF) (Rocca et al., 2019; Sumowski et al., 2013; Sumowski and Leavitt, 2013).

VF, the ability to efficiently generate spoken words in response to specific cues, comprises two subtypes: semantic VF (SVF) and phonemic VF (PVF). SVF relies on lexical retrieval from semantic memory (Delgado-Álvarez et al., 2021; Henry and Crawford, 2004) and is typically assessed by the number of words generated within a semantic category (e.g., ‘animals’). In contrast, PVF relies more on phonemic search strategies and executive functioning, such as inhibitory control (Delgado-Álvarez et al., 2021; Henry and Crawford, 2004), and is assessed using phonemic cues (e.g., the letter ‘s’). Notably, growing evidence suggests that memory and expressive language, the domains predominantly associated with SVF, are among the most affected in contemporary MS cohorts (Brandstadter et al., 2020; Leavitt, 2026; Sumowski et al., 2026). This challenges the longstanding view that information processing speed (IPS) constitutes the central cognitive deficit in MS (Sumowski et al., 2026), motivating a paradigm shift from the still-dominant speed-centric model. Within this evolving perspective, SVF emerges as a particularly informative outcome for understanding cognitive symptoms in MS.

Given this shift, cognitive outcomes reflecting memory and expressive language may also offer a more appropriate framework for investigating reserve-related effects than the traditionally emphasized IPS. Indeed, reports on CR’s moderating effect on IPS are inconsistent: some studies show a moderating role (Benedict et al., 2010; Modica et al., 2016; Sumowski et al., 2009b, 2014) while others fail to replicate this effect, suggesting that CR-related effects may be domain-specific (Ifantopoulou et al., 2019; Nunnari et al., 2016; Rocca et al., 2019; Sumowski et al., 2009a, 2013). Strikingly, meta-analytic evidence indicates that CR consistently moderates the association between structural brain damage and SVF performance in PwMS (Santangelo et al., 2019b), positioning SVF as a particularly appropriate measure for investigating CR-related effects in MS.

Such effects are thought to be expressed through neural efficiency, whereby individuals with greater CR exhibit a more efficient use of available neural resources (Stern, 2012). In MS, functional magnetic resonance imaging (fMRI) evidence suggests that this efficiency is reflected in neural preservation. Specifically, PwMS with higher CR appear to exhibit patterns of functional brain organization that remain closer to those observed in healthy controls despite disease-related effects, a pattern associated with better IPS performance (Fuchs et al., 2019; Sumowski et al., 2010a). Yet, whether a similar pattern of neural preservation underlies CR’s relationship with SVF, the cognitive outcome most consistently benefiting from CR’s protection (Santangelo et al., 2019b), remains unknown.

The rapid and coordinated interaction of distributed semantic and language networks is integral for functions such as SVF (Arrigo et al., 2024; Jefferies, 2013; Ralph et al., 2016). Sustaining this coordination depends on the rhythmic synchronization of neurons across multiple frequency bands, a core mechanism by which brain networks communicate (Fries, 2005; Hipp et al., 2012; Varela et al., 2001). Resting-state magneto−/electroencephalography (rsM/EEG), therefore, offers a direct, physiologically grounded window into this activity, capturing the oscillatory dynamics that underlie neural efficiency. Crucially, alterations to these dynamics have been consistently linked to cognitive dysfunction across neurological conditions (Uhlhaas and Singer, 2006), including MS (De Cock et al., 2023; Keune et al., 2017; Van Der Meer et al., 2013; Schoonhoven et al., 2019; Simon et al., 2023). Given that CR-related cognitive resilience in MS is reflected in preserved brain function (Fuchs et al., 2019; Sumowski et al., 2010a), the preservation of oscillatory activity represents a plausible neurophysiological expression of CR’s protective effects, particularly for SVF.

Although direct evidence linking oscillatory activity with CR in MS is lacking, converging evidence in PwMS and other populations points to the relevance of the alpha (*α*) band. In PwMS, greater alterations in α-band dynamics have been consistently associated with greater deficits across several cognitive domains (Van Der Meer et al., 2013; Van Schependom et al., 2021; Schoonhoven et al., 2019), suggesting that the extent to which *α* activity is preserved matters for maintaining cognitive functioning. Beyond MS, α activity has been linked to CR in individuals with memory complaints (Babiloni et al., 2020; Lopez et al., 2024) and to semantic memory and semantic processing demands, core components of SVF performance, in healthy populations (Doppelmayr et al., 2002; Klimesch, 1999).

Taken together, these observations raise the possibility that preserved α oscillatory activity links CR and SVF in PwMS, implying that these factors may not operate in isolation but rather as components of an interdependent neurocognitive system. This perspective aligns with emerging models of MS that conceptualize cognition as the product of dynamic interactions among cognitive outcomes, protective and risk factors, and their neural substrates (e.g., Bradson et al., 2023; Tsoukaki et al., 2026). Such models may help clarify why PwMS show divergent cognitive profiles, ultimately advancing our understanding of both the disorder and its heterogeneity.

Building on this framework, we tested whether preserved α oscillatory activity links CR and SVF in PwMS. Following a similar approach to Fuchs et al. (2019), preservation was defined as reduced deviations from healthy controls, despite MS-related effects on global spectral power. We hypothesized that higher CR would be associated with preserved α oscillatory activity, which, reflecting CR’s proposed mechanism of neural efficiency and α’s established relevance to CR and semantic processing, would mediate the CR-SVF association. Given that lower α-band (α1; 7.5–10 Hz) alterations have been more consistently linked to attention and memory deficits in MS (Van Schependom et al., 2021; Schoonhoven et al., 2019) and upper α (α2; 10–13 Hz) activity is more prominently implicated in CR and semantic processing, albeit in non-MS populations (Babiloni et al., 2020; Doppelmayr et al., 2002; Klimesch, 1999; Lopez et al., 2024), these sub-bands were quantified separately. Finally, we examined whether the CR → α → SVF relationship varies with physical disability and perceived disease impact to expand on the clinical relevance of our hypothesis.

## Materials and methods

### Participants

Data were obtained from 64 PwMS and 46 age- and sex-matched healthy controls (HC) who were enrolled in the MS-NEUROPLAST clinical trial (ClinicalTrials.gov identifier: NCT04806568) (Styliadis et al., 2024). PwMS were recruited through the Multiple Sclerosis Centre of the 2nd Department of Neurology at AHEPA University Hospital of Thessaloniki, the Department of Neurology at Hippokration General Hospital of Thessaloniki, Greece, and via affiliated private neurologists. The study was approved by the Research Ethics and Deontology Committee of the Aristotle University of Thessaloniki (Protocol no. 254350/2020; approval 1 December 2020) and conducted in accordance with the Declaration of Helsinki. All participants gave written informed consent before participation.

PwMS underwent a neurological screening to determine whether they met the following inclusion criteria: (i) 18–65 years old, (ii) MS diagnosis according to the 2017 Revised McDonald criteria (Thompson et al., 2018), (iii) relapsing–remitting (RRMS) diagnosis as defined by Lublin et al. (2014), (iv) Expanded Disability Status Scale (EDSS) (Kurtzke, 1983) score ranging from 0 to 6.5, (v) neurological stability for at least 1 month prior to inclusion in the study, and (vi) unchanged disease-modifying treatment (DMT) for at least 6 months. The exclusion criteria included: (i) comorbidities of other demyelinating diseases of the central nervous system (CNS), active chronic diseases of the immune system or other significant CNS diseases, (ii) severe psychiatric disorders interfering with compliance or cooperation, and (iii) inability or unwillingness to undergo EEG measurements.

The inclusion criteria for the HC were: (i) normal hearing and (ii) normal or corrected-to-normal vision. The exclusion criteria were: (i) history of neurological, mental, developmental, psychiatric, or physical disorders, (ii) unrecovered neurological disorders (i.e., stroke, traumatic brain injury, etc.), (iii) unstable medication within the last 3 months, (iv) current use of central CNS drugs (e.g., *β*-blockers), (v) concurrent participation in another relevant study, and (vi) inability or unwillingness to undergo EEG measurements.

### Cognitive reserve and neuropsychological battery

CR was assessed using the CR index questionnaire (CRIq), adapted and standardized for the Greek population (Maiovis et al., 2016). CRIq quantifies CR levels in three proxy domains: (i) educational attainment (years of study; CRI-Edu), (ii) occupational complexity (type and years of paid work; CRI-Work), and (iii) engagement in intellectually enriching leisure activities (frequency and years of leisure activities, e.g., reading, socializing, technology use; CRI-Leis). CRIq scores were z-transformed to adjust for the effects of age and sex based on Greek normative data (Maiovis et al., 2016).

Cognitive performance was evaluated using an extensive test battery (see Supplementary materials). In the present study the primary cognitive outcomes are the VF components, with a focus on SVF. VF was assessed with the Verbal Fluency tests (Lezak et al., 2004) measuring phonemic (Chi-Sigma-Alpha, XSA) and semantic (Animals-Fruit-Objects, AFO) tasks. Scores were z-transformed using Greek normative data to adjust for age and education effects (Kosmidis et al., 2004).

Patient-reported outcome measures (PROMs) included fatigue (Modified Fatigue Impact Scale, MFIS) (Fisk et al., 1994), depression (Depression, Anxiety and Stress Scale-21 Items depression subscale, DASS-21ds) (Antony et al., 1998), and disease impact (Multiple Sclerosis Impact Scale-29 Items, MSIS-29) (Hobart, 2001) in PwMS.

### EEG acquisition

rsEEG activity was recorded for 7 min using a high-density Nihon Kohden EEG acquisition system at a sampling rate of 1,000 Hz. Recordings were obtained using two passive 128-channel R-NET caps (Brain Products, size 55 and 57 cm), with electrode impedance maintained below 20 kΩ. All sessions took place in a sound-attenuated, electrically shielded booth at the Medical Physics and Digital Innovation Laboratory, School of Medicine, Aristotle University of Thessaloniki, Greece. Participants sat comfortably, eyes closed, in the dark and were instructed to remain relaxed and minimize movement throughout the recording.

### EEG preprocessing

EEG preprocessing was conducted using the MNE-Python library (Gramfort et al., 2013). The continuous EEG signal was band-pass filtered using a 1 Hz high-pass FIR filter and a 100 Hz low-pass FIR filter. Line noise was removed using a set of five IIR notch filters centered at 50 Hz and its harmonics. The filtered signals were visually inspected to identify channels with poor signal quality.

Independent component analysis (ICA; Infomax algorithm) (Lee et al., 1999) was applied to attenuate ocular, muscular, and other physiological or mechanical artifacts. The number of components was defined to explain 99% of the total signal variance (median *=* 48 components, IQR = 18.5). ICA components were automatically classified using the ICLabel algorithm (Li et al., 2022), and components identified as artifactual with a probability over 70% were removed before reconstructing the cleaned EEG signal (ICA rejected components median *=* 9, IQR = 8.75). The automated classifications were visually inspected to confirm correspondence with typical artifacts.

The reconstructed signal was segmented into subsequent 4-s epochs. Epoch quality was automatically assessed using the Autoreject MNE plugin (Jas et al., 2017), which estimates channel-wise peak-to-peak amplitude thresholds via cross-validation. Rejection and repair parameters were calibrated on a subject-specific basis using the first 20 epochs. Autoreject automatically evaluated multiple rejection-repair strategies, iteratively, allowing interpolation of up to 1, 4, 10, or 15 bad channels per epoch and requiring consensus across 25, 30, 40, or 50% of channels (max 122) to reject an epoch. The optimal parameter combination was selected via cross-validation, after which lower-quality epochs were rejected and remaining bad channels were interpolated. The remaining epochs were evaluated through visual inspection of their power spectral density (PSD) as a final quality-control step, and epochs exhibiting marked deviations from an individual’s average PSD were removed. On average, 5.7 min of data were retained per participant (median *=* 87 epochs; IQR = 20.75).

Additional EEG preprocessing quality-control analyses, including group comparisons of ICA rejection metrics and retained epochs, were conducted to verify that preprocessing did not bias subsequent results, and these analyses are reported in Supplementary Table S1.

### Source estimation

**Figure 1:**
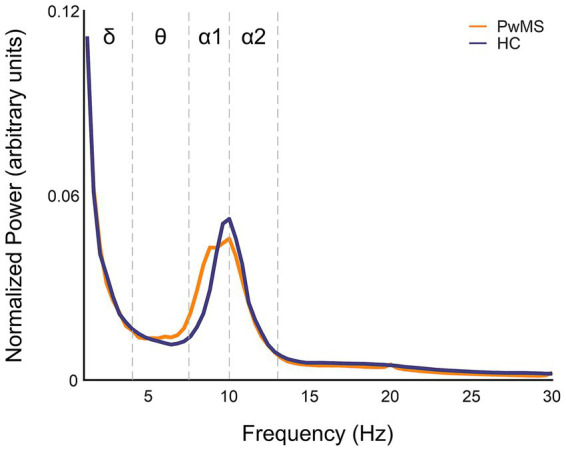
Average sensor-level relative power spectra for PwMS (orange) and HCs (blue). Power spectra were computed per participant using Welch’s method, averaged across epochs and electrodes, normalized by the sum of power across the 1–30 Hz range (relative power), and then averaged within each group. The dashed gray lines represent the thresholds for the frequency bands: *δ* (1–4 Hz), *θ* (4–7.5 Hz), *α*1 (7.5–10 Hz), and *α*2 (10–13 Hz).

The resulting epochs were imported into the LORETA software (v20221229)^1^. The data were transformed to the frequency domain, yielding an average cross-spectrum per participant in the delta (*δ*; 1–4 Hz), theta (*θ*; 4–7.5 Hz), lower alpha (*α*1; 7.5–10 Hz), and upper alpha (*α*2; 10–13 Hz) bands (Figure 1). Canonical frequency bands were adopted in line with the predominant analytical approach in the MS M/EEG spectral power literature (Leocani, 2000; Schoonhoven et al., 2019; Simon et al., 2023), facilitating comparability with existing work. Although the primary hypothesis concerned α-band activity, the δ and θ bands were included due to their established relevance to neuropathology in PwMS (Babiloni et al., 2016; Schoonhoven et al., 2019).

As in Babiloni et al. (2016), current density reconstructions were obtained using the standardized low-resolution electromagnetic tomography (sLORETA) solution (Pascual-Marqui et al., 1994) and projected into source-space based on a realistic MNI-152 head model. The model was restricted to cortical gray matter, as determined by the probabilistic Talairach atlas, and partitioned into 6,239 voxels at a spatial resolution of 5 mm. Cortical activity was normalized by the grand-average current density across all voxels and frequency bands for each participant, yielding relative spectral power values (arbitrary units). For each participant, these values were averaged across all cortical voxels to obtain a single global source-level spectral power per frequency band.

### Statistics

The primary analyses focused on global spectral power differences and the hypothesized relationships among CRIq, spectral power, and VF, including the corresponding mediation model. Global spectral power was selected as the primary measure given its predominant use in MS M/EEG research (De Cock et al., 2023; Keune et al., 2017; Van Der Meer et al., 2013; Schoonhoven et al., 2019; Simon et al., 2023). Regional voxel-wise analyses, ROI-based mediation models, and moderated mediation analyses were considered exploratory. Multiple-comparison correction procedures were applied separately within each analysis family, as described below.

Neuropsychological performance and global spectral power were compared between PwMS and HCs using JASP (version 0.18.3; JASP Team 2025) with independent-sample *t*-tests or Mann–Whitney U tests, depending on normality assessed by the Shapiro–Wilk test. Homogeneity of variance was evaluated using Levene’s test, and Welch’s *t*-tests were applied when equality of variance assumptions were violated. For spectral power analyses, *p*-values were corrected for multiple comparisons using the false discovery rate (FDR) method (Benjamini and Hochberg, 1995) across the four frequency bands. Effect sizes for between-group comparisons were estimated using Cohen’s *d*.

To localize cortical regions contributing to group differences in global spectral power, voxel-wise permutation analyses were performed using statistical nonparametric mapping (SnPM) (Nichols and Holmes, 2002). SnPM was applied to generate an empirical distribution of between-group t-statistics across permutations, conducted specifically for frequency bands exhibiting significant differences at the global level. Voxel-based analyses were conducted for anatomical context and were not included in subsequent regression or mediation models, which focused on global spectral power. Permutation analysis was implemented using the LORETA software.

To facilitate interpretation of inter-individual deviations from HC norms, global spectral power in frequency bands showing significant group differences from HCs was additionally expressed as z-scores relative to the mean and standard deviation of age- and sex-matched HCs. Henceforth, z-score values around 0, indicative of oscillatory patterns approximating HCs, are interpreted as preservation.

Within PwMS, linear and logistic regression models were used to examine associations between CRIq and demographic variables, disease-related characteristics, PROMs, global spectral power (z-scored), and cognitive performance. Models for spectral power and cognitive outcomes were adjusted for age, sex, MS type, disease duration, fatigue, and depression, given their influence on cognitive performance (Langdon et al., 2012). For regression analyses examining associations among CRIq, demographic variables, disease-related measures, spectral power, and cognition, *p*-values were adjusted using FDR correction across all models (minimum 12 associations). When significant associations were observed, follow-up multiple regression analyses including all three CRIq subscales were conducted to identify the most influential CR subdomain. Standardized *β* coefficients were used to describe the magnitude of associations in all regression models. Regression analyses were conducted in JASP.

**Figure 2:**
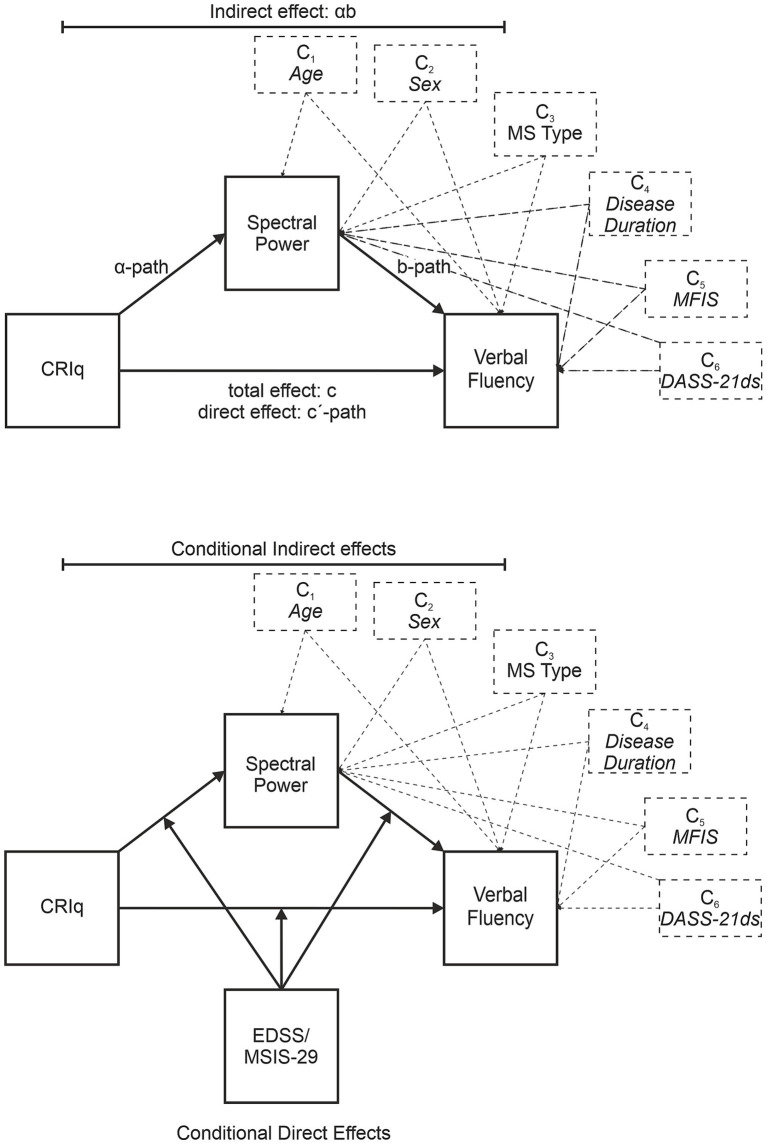
**(A)** Graphical representation of the mediation model illustrating the effects of CRIq (independent variable) on verbal fluency (dependent variable) through global spectral power (mediator), age, sex, MS type, disease duration, fatigue, and depression. The total effect represents the overall CRIq–verbal fluency relationship; the direct effect (c′) reflects the portion independent of spectral power; the indirect effect (αb) quantifies how CRIq influences verbal fluency via the CRIq → spectral power → verbal fluency pathway. The α-path quantifies the CRIq-spectral power association, while the b-path captures the effect of spectral power on verbal fluency. **(B)** Graphical representation of the moderated mediation analysis extending **(A)**, testing whether physical disability (EDSS) or perceived disease impact (MSIS-29 physical and psychological) modifies any of the model paths. Interaction terms are indicated for each path (α, b, and direct). Conditional effects were estimated at low, moderate, and high levels of the moderator (16th, 50th, and 84th percentiles). CRIq, Cognitive Reserve Index questionnaire; MFIS, Modified Fatigue Impact Scale; DASS-21ds, Depression, Anxiety, Stress Scale – 21 Items depression subscale; EDSS, Expanded Disability Status Scale; MSIS-29, Multiple Sclerosis Impact Scale – 29 Items.

To investigate whether spectral power contributes to the CR-VF relationship in PwMS, a mediation analysis was performed using the PROCESS macro model 4 (Hayes, 2013) in SPSS (version 29.0; IBM Corp). CRIq served as the independent variable; the global spectral power (z-scored) in frequency bands significantly associated with both CRIq and cognitive performance served as potential mediators; and cognitive performance scores, which were significantly correlated with CRIq, served as dependent variables (Figure 2A). Covariates were the same as mentioned above. Indirect effects were estimated using percentile bootstrapping (10,000 iterations) and were considered significant when the 95% confidence interval (CI) excluded zero. To account for heteroscedasticity, heteroscedasticity-consistent standard errors were applied. When the overall mediation model was significant, *post-hoc* mediation analyses were conducted for each CRI subscale to identify the primary contributors to the effect.

To examine whether the observed mediations in PwMS were disease-specific, the same mediation models were applied to the HC group. These models included the same predictors and outcomes but were adjusted for covariates available in this group (i.e., age, sex, fatigue, and depression). Results are reported in the Supplementary materials.

To explore the regional signature of the mediation effect, additional exploratory analyses were conducted using spectral power extracted from cortical regions of interest (ROIs). Following a similar approach to Babiloni et al. (2016), ROIs were defined according to Brodmann areas and grouped into frontal (BA 8–11, 44–47), central (BA 1–4, 6), parietal (BA 5, 7, 30, 39, 40, 43), temporal (BA 20, 21, 22, 37, 38, 41, 42), and occipital (BA 17, 18, 19) regions in each hemisphere. Associations between ROI spectral power, CRIq, and SVF were first examined, and ROIs showing associations with both variables were subsequently tested as mediators in separate models. Given the number of regional mediation models tested and the use of a bootstrap-based framework, to account for multiple comparisons, indirect effects were evaluated using 99% bootstrap CIs. Detailed results are provided in the Supplementary materials.

Lastly, to explore the mediation effects in the context of physical disability and disease impact, moderated mediation analyses were conducted using the PROCESS macro model 59 (Hayes, 2013) in SPSS (Figure 2B). EDSS or MSIS-29 (physical and psychological) served as moderators of all mediation paths. The analyses estimated conditional direct and indirect effects across disability or disease impact levels (low, moderate, high), centered on the 16th, 50th, and 84th percentiles. Covariates, bootstrapping procedures, and heteroscedasticity corrections were the same.

Unless otherwise specified, statistical significance was defined as *p <* 0.05 (two-tailed).

## Results

### Population characteristics

Participant characteristics are summarised in Table 1. The groups were matched for age and sex, though PwMS had fewer years of education (3.6 on average; *p <* 0.001, *d* = 0.57). PwMS showed lower total CRIq (*p <* 0.001, *d* = 0.72), and lower scores on the education (*p* = 0.002, *d* = 0.61), work activity (*p* = 0.002, *d* = 0.35), and leisure activities (*p <* 0.001, *d* = 0.40) subscales. They performed worse on both verbal fluency tasks (SVF (AFO test): *p <* 0.001, *d* = 0.85; PVF (XSA test): *p <* 0.001, *d* = 0.42) and reported higher fatigue (*p* = 0.002, *d* = 0.60) and depression (*p* = 0.014, *d* = 0.27) than HCs.

**Table 1:** Demographics, disease-related information, cognitive performance, and responses to PROMs from PwMS and HC.

Characteristic	PwMS (*N =* 64)	HC (*N =* 46)	*p*
Demographics			
Age (years)	41.64 (12.20)	38.85 (11.36)	0.231
Sex (women)	52 (81.25%)	33 (71.74%)	0.240
Education (years)^a^	16 (4)	18 (2.75)	**<0.001**
Disease-related information			
MS subtype (RRMS/PMS)	47/17 (73.4/26.6%)	–	–
DMT (LE/HE/None/Unspecified)	15/40/7/2 (62.5/10.9/3.1/23.5%)	–	–
EDSS	3.02 (1.65)	–	–
Disease Duration (years)^a^	7.5 (10.25)	–	–
Cognitive performance per assessment (z-scored)			
CRIq	−0.27 (0.73)	0.28 (0.82)	**<0.001**
CRI-Edu	0.18 (0.76)	0.67 (0.86)	**0.002**
CRI-Work	−0.27 (0.95)	0.10 (0.62)	**0.002**
CRI-Leis	−0.56 (0.97)	−0.01 (0.74)	**<0.001**
PVF (XSA)	−0.55 (1.15)	0.16 (0.77)	**<0.001**
SVF (AFO)	−0.07 (0.98)	0.77 (0.99)	**<0.001**
PROMs			
MFIS	34.67 (19.13)	25.15 (12.04)	**0.002**
DASS-21ds^a^	4 (7.50)	1 (3)	**0.014**
MSIS-29	54.92 (18.59)	–	–

Sex and MS subtypes are indicated as *N* (%). The mean and standard deviation are presented for most of the remaining variables, whereas ^a^denotes cases in which the median and interquartile range are presented. Significant differences between the groups (*p <* 0.05) are denoted in bold. RRMS, Relapsing–Remitting MS; PMS, Progressive MS; EDSS, Expanded Disability Status Scale; DMT, Disease Modifying Treatment; LE, Low Efficacy; HE, High Efficacy; CRIq, Cognitive Reserve Index questionnaire; CRI-Edu, Cognitive Reserve Index questionnaire – Education subscale; CRI-Work, Cognitive Reserve Index questionnaire – Work Activity subscale; CRI-Leis, Cognitive Reserve Index questionnaire – Leisure activities subscale; PVF, Phonemic Verbal Fluency; XSA, Chi-Sigma-Alpha Phonemic Verbal Fluency Test; SVF, Semantic Verbal Fluency; AFO, Animals-Fruit-Objects Semantic Verbal Fluency Test; PROMs, Patient/Participant-Reported Outcome Measures; MFIS, Modified Fatigue Impact Scale; DASS-21ds, Depression, Anxiety, Stress Scale – 21 Items depression subscale; MSIS-29, Multiple Sclerosis Impact Scale – 29 Items.

### Global spectral power

**Figure 3:**
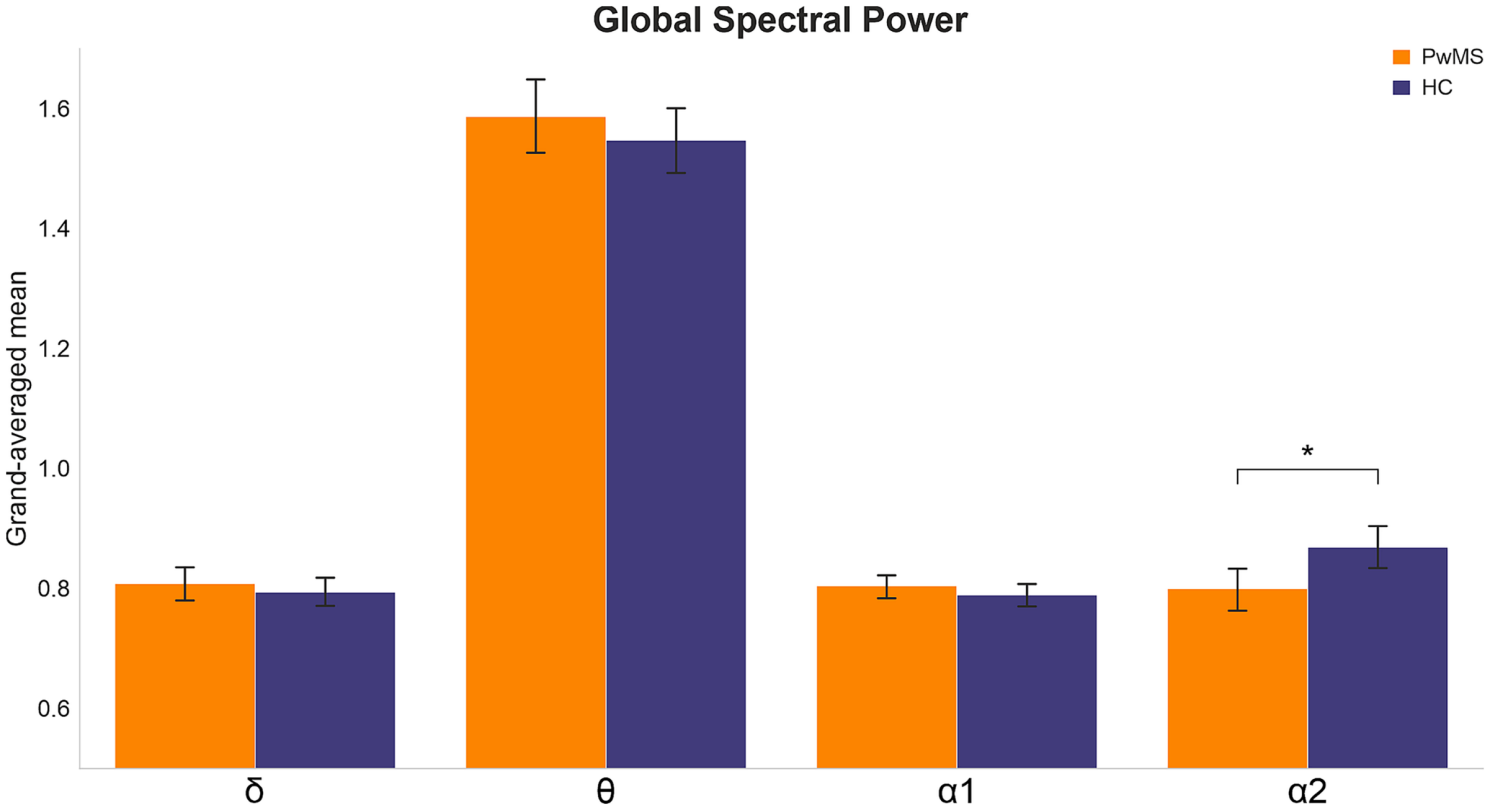
Grand-averaged global spectral power in the δ, θ, α1, and α2 frequency bands for PwMS and HC. The error bars indicate the standard deviation for each group, while * signifies a significant difference between groups with *p <* 0.05. *p*-values were adjusted for multiple comparisons via the FDR method.

Group comparisons revealed significantly reduced global α2 power in PwMS versus HC (t = −2.65, adjusted (adj.) *p* = 0.037, *d* = 0.52). Figure 3 displays the grand-averaged global relative power across the four frequency bands per group.

To assess whether the observed group difference in α2 power was influenced by differences in education, a sensitivity analysis was performed using linear regression with α2 power as the dependent variable and group (PwMS vs. HC) and education as predictors (Supplementary Table S2). In this model, education was not associated with α2 power. Moreover, the Group × Education interaction was non-significant, indicating that the relationship between education and α2 power did not differ between PwMS and HC.

When α2 power was expressed as z-scores relative to healthy controls, 37.5% of PwMS exhibited values below −1 standard deviation (SD), 56.3% fell within ±1 SD, and 6.3% exceeded +1 SD. Stratification by CR (median split) indicated that PwMS with higher CRIq scores were less frequently below −1 SD (31.3% vs. 43.8%) and more frequently within the normative range than those with lower CRIq.

### Regional spectral power

**Figure 4:**
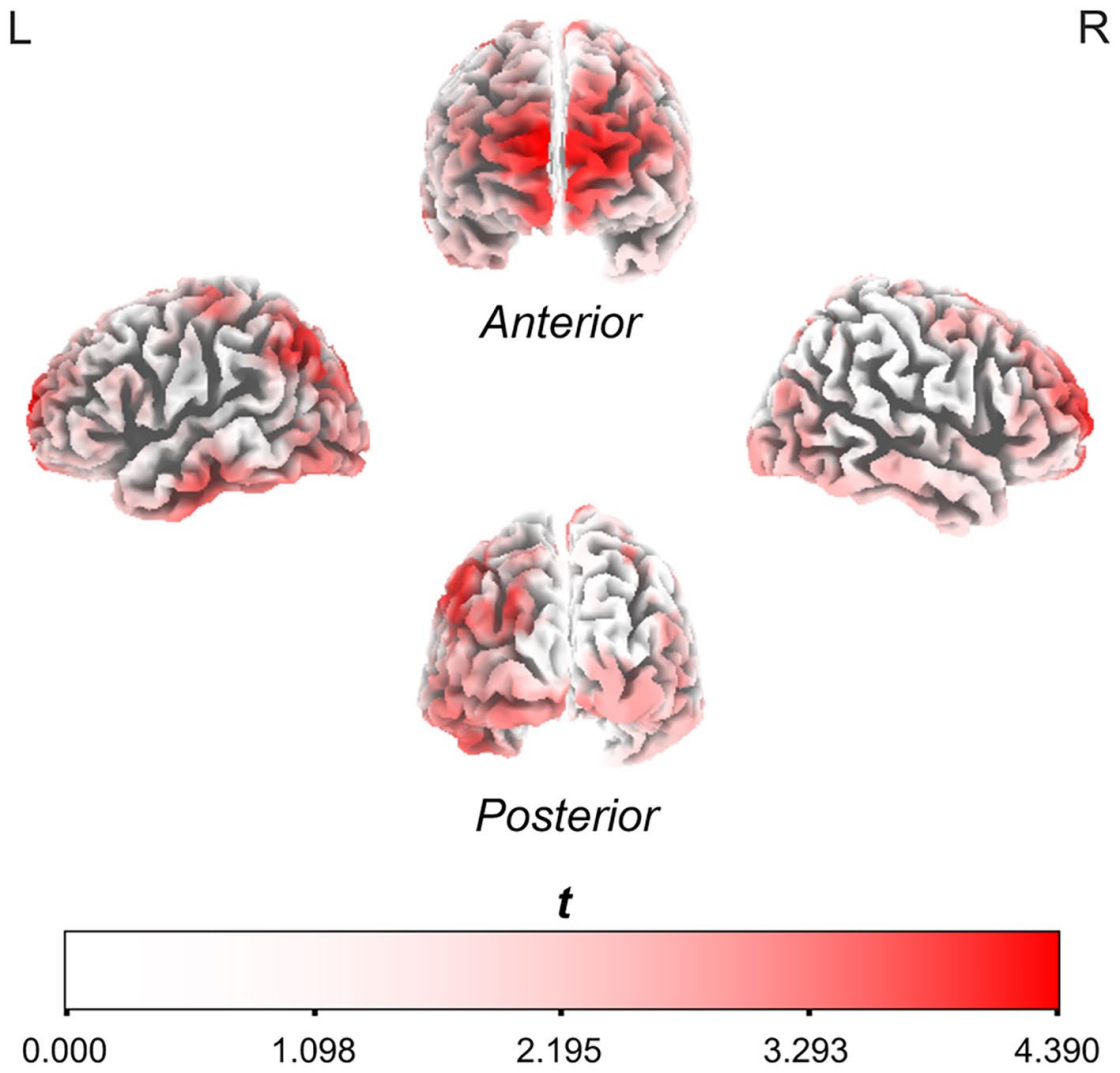
Axial, sagittal, and coronal views of the differences between HC and PwMS in terms of grand-averaged regional spectral power in the α2 band at *p <* 0.05 at t-threshold of 3.029. The L and R represent the left and right hemispheres, respectively. The colour scale represents *t*-values, with positive *t*-values indicating decreased activations in PwMS. The coronal view shows the anterior (top) and posterior (bottom) of the brain.

The permutation analysis of α2 regional spectral power identified significant group differences (HC *vs.* PwMS) in 1237 voxels (*p <* 0.05; Figure 4). Differences were more pronounced in the left hemisphere and involved superior and medial frontal regions, inferior and superior parietal areas, inferior and middle temporal regions, middle occipital areas, and the fusiform gyrus. Full details on Brodmann areas, cluster sizes, *T*-values, and peak coordinates are provided in Supplementary Table S3.

### CRIq correlates

Associations between CRIq and demographics, disease-related variables, PROMs, global power, and cognition are summarised in Table 2 and Supplementary Table S4. CRIq differed by sex (*β* = 1.360, adj. *p* = 0.016 < 0.05), mainly reflecting higher leisure activities-derived CR in women (*β* = 0.986, *p* = 0.034). CRIq correlated strongly with education (*β* = 0.561, adj. *p <* 0.001). CRIq scores were lower in participants with progressive MS type (*β* = −0.713, *p* = 0.029) and among participants with higher depression (*β* = −0.221, *p* = 0.027), although these associations did not remain significant after FDR correction (across 12 models; adj. *p* = 0.058, and adj. *p* = 0.058, respectively). CR was positively correlated with global α2 spectral power (*β* = 0.451, adj. *p* = 0.007) and SVF (AFO test) (*β* = 0.323, adj. *p* = 0.025). This was also reflected in the distribution of α2 z-scores, where PwMS with higher CRIq scores were less frequently below −1 SD and more often within the normative range. The relationship with α2 power was driven by leisure activities-derived CR (*β* = 0.213, *p* = 0.020), whereas the SVF association reflected overall CR, with positive trends for both education (*β* = 0.197, *p* = 0.073) and leisure activities (*β* = 1.360, *p* = 0.078).

**Table 2:** Association between CRIq and demographics, disease-related information, PROMs, global relative power, and verbal fluency performance at *p <* 0.05, adjusted for multiple comparisons via the FDR method for PwMS.

Variable	CRIq			CRI-Edu		CRI-Work		CRI-Leis	
	*β*	*p*	*adj. p*	*β*	*p*	*β*	*p*	*β*	*p*
Demographics									
Age	−0.171	0.177	0.236	–	–	–	–	–	–
Sex^a^	1.360	0.004	**0.016**	−0.171	0.605	0.683	0.123	0.986	**0.034**
Education	0.561	<0.001	**<0.001**	0.676	**<0.001**	0.040	0.521	0.135	0.151
Disease-related information									
MS type^a^	−0.713	0.029	0.058	–	–	–	–	–	–
EDSS	−0.156	0.219	0.262	–	–	–	–	–	–
Disease Duration	−0.148	0.244	0.266	–	–	–	–	–	–
PROMs									
MFIS	−0.221	0.079	0.118	–	–	–	–	–	–
DASS-21ds	−0.277	0.027	0.058	–	–	–	–	–	–
MSIS-29	−0.245	0.051	0.087	–	–	–	–	–	–
Global spectral power^b^									
α2	0.451	0.001	**0.007**	0.102	0.411	0.158	0.213	0.323	**0.020**
Cognitive performance^b^									
PVF (XSA)	−0.019	0.951	0.951	–	–	–	–	–	–
SVF (AFO)	0.323	0.008	**0.025**	0.197	0.073	0.030	0.788	0.212	0.078

When a significant association between a variable and CRIq occurred, the relation between the same variable and the CRIq subscales was also investigated. Bold type denotes significance. ^a^Indicates that the logistic regression was applied. ^b^Indicates regression models adjusted for age, sex, MS type, disease duration, MFIS, and DASS-21ds. CRIq, Cognitive Reserve Index questionnaire; CRI-Edu, Cognitive Reserve Index questionnaire – Education subscale; CRI-Work, Cognitive Reserve Index questionnaire – Work activity subscale; CRI-Leis, Cognitive Reserve Index questionnaire – Leisure activities subscale; adj, adjusted; EDSS, Expanded Disability Status Scale; PROMs, Patient/Participant-Reported Outcome Measures; MFIS, Modified Fatigue Impact Scale; DASS-21ds, Depression, Anxiety, Stress Scale – 21 Items depression subscale; MSIS-29, Multiple Sclerosis Impact Scale – 29 Items; PVF, Phonemic Verbal Fluency; XSA, Chi-Sigma-Alpha Phonemic Fluency Test; SVF, Semantic Verbal Fluency; AFO, Animals-Fruit-Objects Semantic Fluency Test.

### Mediation and moderated mediation analyses

**Figure 5:**
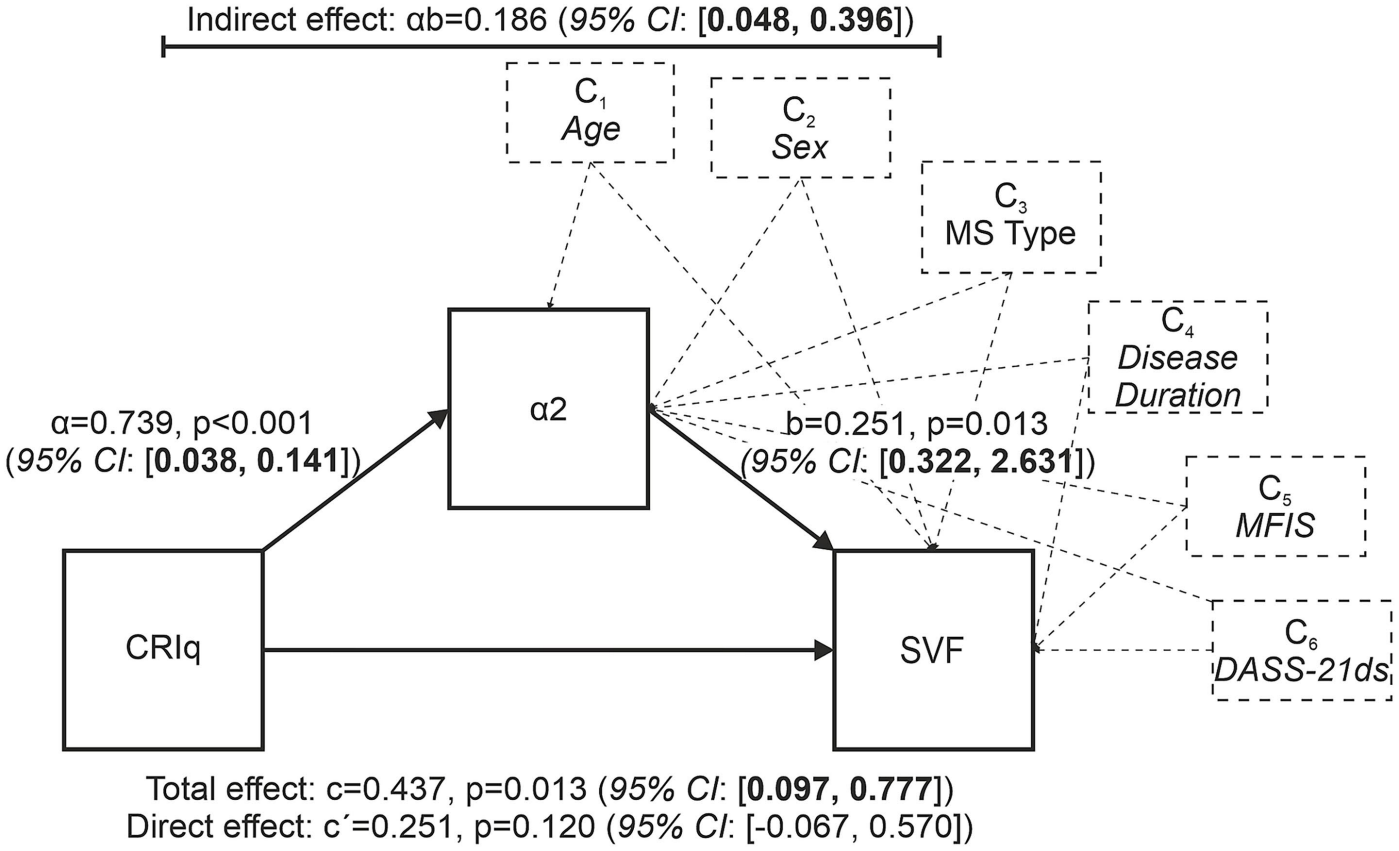
Statistical diagram of the effects of the mediation analysis with CRIq as the independent variable, global α2 spectral power as the mediator, performance on SVF as the dependent variable, and age, sex, MS type, disease duration, MFIS, and DASS-21ds as covariates. The mediation analysis explores three different paths: total (c, which indicates the relationship between CRIq and SVF while ignoring the effect of global α2 spectral power), direct (c’, which indicates if the relationship between CRIq and SVF is independent of the effect of global α2 spectral power), and indirect (ab, which indicates whether CRIq’s influence on global α2 spectral power can be transmitted to SVF through the CRIq → α2 → SVF path). Alternatively, the indirect effect can be estimated as the product of paths α and b (hence denoted as αb). In our model, the total (c = 0.437, 95% CI: [0.097, 0.777], *p* = 0.013 < 0.05) and indirect (αb = 0.186, 95% CI = [0.048, 0.396], *p <* 0.05) effects are significant, indicating that the relationship between CRIq and SVF is dependent on global α2 spectral power, as CR appears to exert influence on SVF primarily through α2, i.e., individuals with higher CR have higher α2 spectral power and thus higher performance on SVF. CI, Confidence Interval; CRIq, Cognitive Reserve Index questionnaire; SVF, Semantic Verbal Fluency; MFIS, Modified Fatigue Impact Scale; DASS-21ds, Depression, Anxiety, Stress Scale – 21 Items – depression subscale.

The mediation analysis results examining the relationships among CRIq, global α2 spectral power, and SVF are summarised in Figure 5, Table 3, and Supplementary Table S5. Additional analyses examined the contributions of individual CRIq subscales (Table 3 and Supplementary Tables S6, S7), whether the effect was present in HCs (Supplementary Tables S12, S13), its regional distribution across cortical ROIs (Supplementary Tables S14–S19), and its potential modulation by disease severity (Table 4 and Supplementary Tables S20–S22). It should be noted that although mediation analysis decomposes total effects into direct and indirect components, it does not establish causality. The present findings should therefore be interpreted as associations between the examined variables.

**Table 3:** Mediation analysis of CRIq, α2 spectral power, and semantic verbal fluency in PwMS.

Effect	Path	Coeff.		SE	*t*	95% CI		*p*
						Lower	Upper	
Total	CRIq → SVF	*c*	0.437	0.170	2.572	**0.097**	**0.777**	**0.013**
Direct	CRIq → SVF	*c΄*	0.251	0.159	1.580	−0.067	0.570	0.120
Indirect	CRIq → α2 → SFV	*ab*	0.186	0.090	–	**0.045**	**0.390**	–
Total	CRI-Edu → SVF	*c*	0.276	0.155	1.778	−0.035	0.587	0.081
Direct	CRI-Edu → SVF	*c΄*	0.216	0.137	1.572	−0.064	0.476	0.122
Indirect	CRI-Edu → α2 → SVF	*ab*	0.060	0.070	–	−0.058	0.217	–
Total	CRI-Leis → SVF	*c*	0.232	0.165	1.409	−0.098	0.563	0.164
Direct	CRI-Leis → SVF	*c΄*	0.115	0.165	0.667	−0.230	0.460	0.508
Indirect	CRI-Leis → α2 → SVF	*ab*	0.118	0.058	–	**0.026**	**0.252**	–

The bold type denotes significance, determined by CIs that exclude 0 (as defined by the bootstrap-derived 95% CI lower and upper bounds). Coeff., Coefficient; SE, Standard Error; CI, Confidence Interval; CRIq, Cognitive Reserve Index questionnaire; CRI-Edu, Cognitive Reserve Index questionnaire – Education subscale; CRIq-Leis, Cognitive Reserve Index questionnaire – Leisure activities subscale; SVF, Semantic Verbal Fluency test.

**Table 4:** Conditional indirect effects of the moderated mediation model at low, moderate, and high levels of EDSS and MSIS-29 (Physical and Psychological), adjusted for age, sex, MS type, disease duration, MFIS, and DASS-21ds.

Moderator		Coeff.	SE	*t*	95% CI	
					Lower	Upper
Conditional indirect effects of CRIq on SVF through α2 spectral power						
EDSS	1.2	0.157	0.129	–	−0.018	0.478
	3	0.186	0.097	–	**0.036**	**0.412**
	5	0.221	0.183	–	−0.005	0.681
MSIS-29 Physical	22.4	0.131	0.124	–	−0.012	0.455
	35	0.168	0.096	–	**0.031**	**0.401**
	53.2	0.225	0.143	–	**0.011**	**0.565**
MSIS-29 Psychological	11.4	0.117	0.113	–	−0.027	0.396
	17	0.162	0.094	–	**0.020**	**0.384**
	23.6	0.220	0.149	–	**0.014**	**0.594**

The bold type denotes significance, determined by CIs that exclude 0 (as defined by the bootstrap-derived 95% CI lower and upper levels). The low levels of the moderators are centred around the 16^th^ percentile, the moderate levels around the 50^th^ percentile, and the high levels around the 84^th^ percentile. Coeff, Coefficient; SE, Standard Error; EDSS, Expanded Disability Status Scale; MSIS-29, Multiple Sclerosis Impact Scale-29 items; CRIq, Cognitive Reserve Index questionnaire; SVF, Semantic Verbal Fluency; MFIS, Modified Fatigue Impact Scale; DASS-21ds, Depression, Anxiety, Stress Scale-21 items depression subscale; SE, Standard Error; CI, Confidence Interval.

A significant total effect of CRIq on SVF (AFO test) was found (c = 0.437, *p* = 0.013). The direct path was non-significant (c΄ = 0.251, *p* = 0.120), indicating that this relationship is not entirely independent of α2 oscillatory activity. The indirect effect (CRIq→α2 → SVF; αb = 0.186, 95% CI = [0.048, 0.396]) was significant, indicating that global α2 spectral power statistically mediates the CR-SVF relationship. The two individual paths (Figure 5) showed that higher CR was associated with higher α2 spectral power (α-path = 0.739, *p <* 0.001), corresponding to values closer to those observed in HC, which, in turn, was related to better SVF performance (b-path = 0.251, *p* = 0.003).

Among the CRIq subscales, only CRI-Edu and CRI-Leis, which showed positive trends with SVF, were further analyzed. For CRI-Edu, the non-significant association with α2 spectral power restricted testing to the direct path, which was also non-significant (c΄ = 0.251, *p* = 0.122). This suggests that the association between CRI-Edu and SVF was not statistically mediated by α2 power, although it may not be entirely independent of α2 dynamics. In contrast, for CRI-Leis, the significant association with α2 spectral power directed the focus to the indirect path (αb = 0.118, 95% CI [0.026, 0.252]), indicating an indirect association between CRI-Leis and SVF through α2 oscillatory activity.

Years of education were not included as a covariate in the primary mediation models, as education constitutes a core component of CR and is explicitly incorporated into CRIq (Nucci et al., 2012). Consequently, adjusting for education would remove variance that is intrinsic to the reserve construct itself. Nevertheless, to evaluate whether the observed group difference in years of education between PwMS and HC influenced the mediation results, a sensitivity analysis was conducted by rerunning the CRIq→α2 → SVF mediation model with years of education included as a covariate. The indirect effect remained significant (αb = 0.222, 95% CI [0.049, 0.456]; Supplementary Table S8), indicating that educational differences between groups do not account for the observed CRIq→α2 → SVF effect.

No significant mediation effect was observed in the HC group, as neither the total effect of CR on SVF nor the indirect effect via α2 spectral power reached significance (Supplementary Tables S12, S13). These findings suggest that the CR → α2 → SVF effect may be specific to PwMS.

Exploratory ROI analyses were conducted to assess the regional specificity of the observed mediation effect. Across ROIs, α2 spectral power was positively associated with CRIq, whereas only frontal, central, occipital, and right parietal regions showed significant associations with SVF after correction for multiple comparisons. ROI-specific mediation analyses revealed significant indirect effects in the left frontal (αb = 0.178, 99% CI [0.011, 0.465]), left central (αb = 0.166, 99% CI [0.007, 0.421]), right frontal (αb = 0.219, 99% CI [0.020, 0.516]), and right parietal (αb = 0.216, 99% CI [0.013, 0.511]) regions.

Moderated mediation models tested whether CR’s indirect effects on SVF varied relative to physical disability (EDSS) or disease impact (MSIS-29 Physical and Psychological). None of the interaction terms were significant (see Supplementary Tables S20–S22), indicating no formal evidence of moderation of the individual paths. However, based on reporting recommendations, even in the absence of significant moderation, conditional indirect effects may still be examined and interpreted, albeit cautiously (Hayes, 2013) (Table 4). In our case, the conditional indirect effects indicated that the CRIq → α2 → SVF effect differed significantly from zero only at moderate levels of physical disability (EDSS centered at 3, αb = 0.186, 95% CI [0.036, 0.412]), but not at low or high levels. In contrast, when the MSIS-29 subscales served as moderators, the indirect effect differed significantly from zero at moderate levels of physical and psychological impact (MSIS-Physical centered at 35, αb = 0.168, 95% CI [0.031, 0.401]; MSIS-29 Psychological centered at 17, αb = 0.162, 95% CI [0.020, 0.384]), as well as at high levels of disease impact (MSIS-Physical centered at 53.2, αb = 0.225, 95% CI [0.011, 0.565]; MSIS-29 Psychological centered at 23.6, αb = 0.220, 95% CI [0.014, 0.594]).

## Discussion

This study examined whether preserved α oscillatory activity (i.e., spectral power closer to that of HCs) contributes to the association between CR and SVF in PwMS. We found that α2 activity mediated the relationship between CR and SVF, while no such pattern was observed in HCs. Higher CR was associated with preserved α2 activity, which, in turn, was linked to better SVF performance. No corresponding associations were observed for PVF. Exploratory moderated mediations were not significant; nevertheless, conditional indirect effects suggested that the CR → α2 → SVF path may be most evident at moderate levels of physical disability and moderate-to-high levels of perceived disease impact.

The present findings contribute to a growing reconsideration of the cognitive domains most relevant to resilience in MS. Although cognition in MS has historically been viewed through the lens of IPS, reserve-related effects on IPS have been conflicting, whereas associations with SVF have emerged more consistently (Santangelo et al., 2019b). Growing evidence suggests that memory and expressive language are central features of the contemporary cognitive phenotype in MS (Brandstadter et al., 2020; Leavitt, 2026; Sumowski et al., 2026), making SVF a particularly relevant outcome. Understanding resilience in MS requires frameworks linking clinically relevant cognitive outcomes to their underlying neural mechanisms. To our knowledge, this is the first study in MS to jointly examine CR, oscillatory activity, and SVF performance, providing a more mechanistic account of reserve-related resilience at the neural level.

These findings extend previous fMRI-based evidence linking higher CR to more neurotypical brain function in PwMS (Fuchs et al., 2019; Sumowski et al., 2010a), suggesting that such reserve-related preservation may also be reflected in oscillatory dynamics. Building on evidence that CR moderates the impact of structural damage on SVF (Santangelo et al., 2019b), our results implicate preserved α2 activity as a neurophysiological component of CR-related resilience in semantic-language functions. This interpretation is consistent with converging evidence from non-MS populations linking α2 oscillations to CR in individuals with memory complaints (Babiloni et al., 2020, 2021; Lopez et al., 2024), and to semantic processing demands and semantic memory in healthy individuals (Doppelmayr et al., 2002; Klimesch, 1999). Our findings extend this literature to MS, while the absence of a comparable pattern in HCs suggests that this mediation may be particularly relevant in the context of MS-related pathology.

Regional analyses provided further insight into the neural systems through which CR-related resilience may be expressed in relation to semantic-language functioning. Significant indirect effects emerged in bilateral frontal, left central, and right parietal regions, implicated in lexical-semantic search and control processes (Arrigo et al., 2024; Jefferies, 2013; Ralph et al., 2016). These regions overlap with the frontoparietal network, a hub-like system supporting coordination across distributed brain networks (Marek and Dosenbach, 2018) and thought to contribute to CR-related resilience to brain aging and pathology (Stern et al., 2019; Varela-López et al., 2022). Preserved α2 activity in these areas may therefore reflect the maintenance of large-scale network interactions supporting efficient lexical-semantic retrieval during SVF performance (Arrigo et al., 2024).

Although both VF subtypes were impaired in our sample, consistent with VF being a sensitive measure of cognitive dysfunction in MS (Henry and Beatty, 2006; Leavitt et al., 2025), only SVF was significantly associated with CR. This aligns with evidence that CR preferentially protects functions dependent on long-term memory systems, including semantic memory processes central to SVF (Rocca et al., 2019; Sumowski et al., 2013; Sumowski and Leavitt, 2013). By contrast, PVF relies more heavily on inhibitory control and IPS (Delgado-Álvarez et al., 2021; Henry and Crawford, 2004; Lebkuecher et al., 2021), domains that may be less susceptible to CR-related benefits. This pattern mirrors previous findings that CR has stronger effects on memory than IPS (Sumowski et al., 2009a).

A plausible explanation for why SVF, and not PVF, was associated with α2 activity lies in their distinct neural substrates. In PwMS, SVF has been linked to the thalamus, whereas PVF has been associated with the caudate nucleus (Matías-Guiu et al., 2018). This distinction is particularly relevant given the central role of thalamic pathology in MS, with thalamic atrophy representing a robust marker of disease progression and cognitive dysfunction (Minagar et al., 2013; Schoonheim et al., 2015a). The thalamus is also critically involved in the generation and synchronization of α oscillations (Halgren et al., 2019; Hughes and Crunelli, 2005), and thalamic atrophy has been linked to alterations in *α*-band activity in PwMS (Van Schependom et al., 2021; Schoonhoven et al., 2019). Together, these observations suggest α2 activity may be preferentially linked to cognitive processes underlying SVF rather than PVF. Consistent with this interpretation, previous rsM/EEG studies have reported no association between α2 power and PVF (Nauta et al., 2024; Van Schependom et al., 2021).

These associations may also have implications for interpreting electrophysiological markers of cognitive dysfunction in MS. Neuronal slowing, typically indexed by increased low-frequency power and reduced high-frequency activity, is widely regarded as a marker of global cognitive decline in MS. However, the functional specialization of individual frequency bands may provide information beyond overall cognitive status. For instance, α1 oscillations have been consistently linked to attention and episodic memory in neurotypical populations (Klimesch, 1999), domains that are also associated with α1 alterations in MS (Van Schependom et al., 2021; Schoonhoven et al., 2019). In contrast, α2 activity has been more closely linked to semantic processing (Doppelmayr et al., 2002; Klimesch, 1999), consistent with the present findings. Together, these observations suggest that band-specific oscillatory alterations may reflect vulnerability within specific cognitive domains rather than merely signaling global cognitive decline.

Notably, the association between CR and α2 activity was primarily driven by leisure-derived CR, consistent with evidence that leisure activities independently contribute to CR beyond education (Sumowski et al., 2010b). This pattern suggests that α2 activity may be particularly sensitive to experiences that promote reserve accumulation across the lifespan. This observation may have practical relevance, given that interventions designed to enhance CR largely depend on sustained engagement in leisure activities (Sumowski, 2015). Accordingly, the present findings raise the possibility that changes in reserve levels may be accompanied by corresponding changes in oscillatory dynamics.

Although the formal moderated mediation analyses were not statistically significant, examining conditional indirect effects can provide useful insight into whether mediation is present at specific values of the moderator (Hayes, 2013). Here, the indirect effect differed significantly from zero only at moderate levels of physical disability (centered at EDSS = 3) and was non-significant at low (EDSS < 2) and higher levels (EDSS > 4). Similarly, significant indirect effects were observed at moderate-to-high levels of physical and psychological impact. These findings raise the possibility that the contribution of α2 activity to reserve-related resilience may vary across stages of disease burden. Such a pattern is consistent with theoretical accounts that reserve-related adaptive neural processes may require a degree of neural challenge to engage (Stern, 2012; Sumowski and Leavitt, 2013), but may become less effective as disease burden increases (Rocca et al., 2019; Schoonheim et al., 2015b). Nevertheless, given the absence of significant formal moderation, these observations should be considered exploratory pending replication.

Taken together, these findings suggest that CR may support the preservation of neural processes underlying semantic-language function in PwMS. Although causal inferences cannot be drawn, this possibility may be particularly relevant given evidence that semantic-language abilities are affected early in the course of MS (Brandstadter et al., 2020; Sumowski et al., 2026).

Several limitations should be acknowledged. Structural brain information was not included in the present study. Consequently, it was not possible to determine the extent to which the observed associations reflect reserve-related neural processes, structural brain integrity, or their interaction. The absence of MRI data precluded assessment of lesion burden and distribution, factors known to disrupt white-matter conduction and α-band dynamics (Mazzara et al., 2025), and necessitated the use of a standard head model for source reconstruction; this limitation, though, is more relevant to regional specificity than to the primary global spectral outcomes. ROI analyses were based on anatomical Brodmann groupings rather than functionally defined networks, potentially obscuring more localized or network-specific effects. Multimodal replications integrating structural and electrophysiological measures, with finer-grained parcellations and network-based approaches, would help clarify the contribution of structural pathology and strengthen anatomical specificity. The cross-sectional design precludes causal and temporal inferences, therefore findings were interpreted as associations; directional relationships among CR, oscillatory activity, and SVF performance remain to be established. Reliance on a single CR proxy (CRIq) and quantification of SVF performance by total word count alone may limit generalizability; further work should examine additional reserve dimensions and SVF components, such as clustering and switching.

Treatment-specific effects were not adequately evaluated due to the heterogeneity of DMTs. As an initial sensitivity analysis, DMTs were grouped by efficacy class (see Supplementary materials) and included as a covariate in the primary mediation model; the mediation remained significant (Supplementary Table S9). However, the available sample did not permit examination of individual DMTs, which may differentially influence neuroinflammatory processes, cognition, and brain function. Sex-related effects similarly merit attention, given known differences in disease progression (Voskuhl and Gold, 2012), functional organization (Schoonheim et al., 2012) in MS, and engagement in cognitively enriching activities observed here. Although sex was included as a covariate, exploratory analyses conducted separately in women and men revealed a significant indirect effect only in women (Supplementary Tables S10, S11). However, the small sample size of men (*N =* 12) precludes meaningful interpretation of this apparent difference.

## Conclusion

In conclusion, our findings implicate α2 oscillatory activity in reserve-related resilience in PwMS. α2 power mediated the relationship between CR and SVF, with higher CR associated with preserved α2 activity, which, in turn, was linked to better SVF performance. The absence of a comparable pattern in healthy controls indicates that this effect may be particularly relevant to disease-specific adaptive processes. Collectively, these findings provide a more mechanistic account of reserve-related resilience in MS and highlight semantic-language function as a key domain through which such resilience may be expressed.

## Data Availability

The raw data supporting the conclusions of this article will be made available by the authors, without undue reservation.

## Footnotes

1
www.uzh.ch/keyinst/loreta.htm

## Author’s note

All copyrighted scales and questionnaires used in this study were employed with appropriate permission from the respective copyright holders. Material from the SDMT copyright © 1973, by Western Psychological Services. Reproduced by C Styliadis, PhD, Aristotle University of Thessaloniki (CON1281), for specific, limited research use, under license of the publisher, WPS (rights@wpspublish.com). No additional reproduction, in whole or in part, by any medium or for any purpose, may be made without the prior, written authorization of WPS. All rights reserved. The MMSE, BVMT-R, and SNST were initially administered in this study using Greek translations not authorized by PAR. This has since been formally rectified through licensing, following proactive outreach by the research team to PAR. The MMSE, BVMT-R and SNST are copyrighted instruments and may not be used or reproduced in whole or in part, in any form or language, or by any means without written permission of PAR.
