# Supplementary material for: Preserved α2 oscillations underpin reserve-related resilience of semantic verbal fluency in multiple sclerosis
Source: Front Neurosci. 2026 Jul 22;20:1869738. doi: 10.3389/fnins.2026.1869738 (PMC13438199; doi:10.3389/fnins.2026.1869738)
Supplement: Supplementary file 1 [file Supplementary_file_1.docx]

# ***Supplementary Material***

## **Disease-Modifying-Treatments**

DMTs were classified as high- or low-efficacy. High efficacy DMTs include fingolimod (N = 12), ofatumumab (N = 6), alemtuzumab (N = 2), cladribine (N = 6), ocrelizumab (N = 6), and natalizumab (N = 12). Low efficacy DMTs include teriflunomide (N = 1), interferon beta-1a (N = 1), glatiramer acetate (N = 5), Siponimod (N = 1), and dimethyl fumarate (N = 7).

## **Neuropsychological battery**

All participants underwent neuropsychological assessments, validated in the Greek population and conducted by a certified neuropsychologist, to evaluate their cognitive state. The participants completed the Brief International Cognitive Assessment for MS (BICAMS) battery (Benedict et al., 2012), comprising the Greek Verbal Learning and Memory Test (GVLT), revised Brief Visuospatial Memory Test (BVMT-R), and Symbol Digit Modalities Test-Oral form (SDMT-Of). The participants also completed the Stroop Neuropsychological Screening Test (Trenerry et al., 1989) (SNST), the Wechsler Adult Intelligence Subscales (WAIS-IV) of Digit Span [DS, forward (DSf), backward (DSb), and sequencing (DSs)] (Wechsler, 2008), and the Verbal Fluency tests that measure semantic [Animals-Fruit-Objects (AFO)] and phonemic [Chi-Sigma-Alpha (XSA)] fluency (Lezak et al., 2004). All assessment scores were converted to z-scores based on normative data from the literature for the Greek population (Kosmidis et al., 2004; Messinis et al., 2021; Vlahou et al., 2013; Wechsler, 2014; Zalonis et al., 2009), to adjust for the effects of age and education, and in some cases sex (e.g., GVLT and BVMT-R).

## **EEG Preprocessing Quality Control**

S1. Descriptive statistics, group comparisons, and effect sizes (Cohen’s d) for EEG preprocessing parameters in PwMS and HCs.

|  | **MS** | **HC** | ***d*** | ***p*** |
| --- | --- | --- | --- | --- |
| ICA components | 46 (19) | 53.5 (18.75) | 0.422 | **0.031** |
| ICA rejected components | 8 (8.25) | 10 (8) | 0.154 | 0.169 |
| ICA rejection percentage | 19.38 (16.25) | 22.22 (12.08) | 0.104 | 0.355 |
| Retained epochs | 87 (20.25) | 86 (17) | 0.017 | 0.882 |

*Data is presented as median (IQR). The ICA rejection percentage was calculated as (ICA rejected components / ICA components) × 100. Group comparisons were performed using independent-samples t-tests or Mann-Whitney U tests, depending on the normality of the data. Bold type denotes significance. HC exhibited a higher number of estimated ICA components, but no group differences were observed in either the number or percentage of rejected components. No group differences were observed in retained epochs following preprocessing. Abbreviations: PwMS, People with MS; HCs, Healthy Controls; ICA, Independent Component Analysis*

## **Sensitivity Analysis**

S2. Sensitivity analyses examining the contribution of education to group (PwMS vs HCs) differences in global α2 spectral power.

| **Predictor** | **α2 ~ Group + Education** | | | **α2 ~ Group + Education + Group × Education** | | |
| --- | --- | --- | --- | --- | --- | --- |
|  | B | SE | p | B | SE | p |
| Intercept | 0.796 | 0.065 | **<0.001** | 0.741 | 0.101 | **<0.001** |
| Group | 0.068 | 0.003 | **0.025** | 0.211 | 0.146 | 0.152 |
| Education | 0.000 | 0.004 | 0.953 | 0.013 | 0.013 | 0.483 |
| Group × Education | − | − | − | -0.001 | 0.009 | 0.321 |
|  | Adjusted R^2^ = 0.044 | | | Adjusted R^2^ = 0.043 | | |

*Two linear regression models are shown: a main-effects model including group and education, and a second model additionally including the Group × Education interaction. Global α2 spectral power served as the dependent variable in both models. Bold type denotes significance. Group was coded as 1 = PwMS and 2 = HCs. Abbreviations: B, unstandardized regression coefficients; SE, Standard Error; PwMS, People with Multiple Sclerosis; HCs, Healthy Controls.*

## **Differences in regional spectral power between HC and PwMS**

*S3. Significant regional relative power differences between HC and PwMS per band at p<0.05, adjusted for multiple comparisons via the FDR method.*

| **Hemisphere** | **Anatomical Area** | **BA** | **CS** | ***T*** | **MNI coordinates (mm)** | | |
| --- | --- | --- | --- | --- | --- | --- | --- |
|  |  |  |  |  | ***x*** | ***y*** | ***z*** |
| **α2 (10-13 Hz)** | | | | | | | |
| L | ANG | 39 | 15 | 4.043 | -50 | -75 | 30 |
| L | medFG | 10 | 22 | 4.040 | -5 | 65 | 10 |
| L | ITG | 20 | 64 | 4.002 | -40 | -20 | -35 |
|  |  | 37 | 32 | 3.740 | -50 | -70 | -5 |
| L | SFG | 10 | 25 | 3.985 | -15 | 60 | 25 |
|  |  | 11 | 20 | 3.689 | -5 | 65 | -10 |
| L | MTG | 39 | 14 | 3.976 | -50 | -75 | 25 |
|  |  | 21 | 60 | 3.970 | -65 | -20 | -15 |
|  |  | 37 | 16 | 3.869 | -60 | -65 | 5 |
|  |  | 38 | 10 | 3.295 | -35 | 5 | -45 |
| L | IPL | 40 | 32 | 3.961 | -50 | -60 | 45 |
| L | FFG | 20 | 49 | 3.932 | -45 | -20 | -30 |
|  |  | 19 | 22 | 3.669 | -50 | -70 | -20 |
|  |  | 37 | 46 | 3.641 | -55 | -50 | -20 |
| L | SPL | 7 | 14 | 3.916 | -40 | -65 | 50 |
| L | CUN | 19 | 18 | 3.906 | -20 | -90 | 35 |
| L | MOG | 19 | 36 | 3.805 | -55 | -70 | -10 |
|  |  | 18 | 21 | 3.702 | -35 | -90 | 10 |
| L | MFG | 10 | 17 | 3.800 | -20 | 60 | 25 |
| L | PCUN | 19 | 12 | 3.714 | -20 | -85 | 35 |
| L | SOG | 19 | 11 | 3.693 | -40 | -85 | 25 |
| L | PCG | 6 | 12 | 3.671 | -40 | -20 | 65 |
| L | LING | 18 | 21 | 3.651 | -25 | -95 | -10 |
| L | POG | 1 | 15 | 3.585 | -50 | -25 | 60 |
| L | IOG | 18 | 23 | 3.518 | -25 | -90 | -15 |
| L | STG | 38 | 46 | 3.297 | -45 | 20 | -25 |
| L | IFG | 47 | 16 | 3.220 | -40 | 20 | -20 |
| R | SPL | 7 | 11 | 4.095 | 30 | -65 | 50 |
| R | SFG | 10 | 36 | 4.066 | 25 | 60 | 15 |
|  |  | 9 | 16 | 3.697 | 20 | 55 | 30 |
|  |  | 11 | 12 | 3.640 | 10 | 65 | -10 |
|  |  | 6 | 11 | 3.564 | 10 | 10 | 70 |
| R | medFG | 10 | 29 | 4.059 | 5 | 65 | 10 |
| R | MFG | 10 | 19 | 4.006 | 30 | 60 | 15 |
|  |  | 8 | 15 | 3.686 | 35 | 30 | 50 |
|  |  | 6 | 15 | 3.600 | 15 | 10 | 65 |
| R | MOG | 19 | 14 | 3.748 | 55 | -70 | 5 |
|  |  | 18 | 16 | 3.483 | 15 | -100 | 10 |
| R | MTG | 39 | 26 | 3.721 | 45 | -75 | 25 |
|  |  | 37 | 19 | 3.643 | 55 | -65 | 5 |
|  |  | 21 | 21 | 3.613 | 70 | -25 | -10 |
| R | PCL | 5 | 18 | 3.647 | 15 | -45 | 60 |
| R | LING | 18 | 35 | 3.644 | 0 | -95 | -5 |
| R | ITG | 37 | 13 | 3.601 | 50 | -70 | -5 |
|  |  | 20 | 15 | 3.337 | 65 | -20 | -20 |
| R | PCG | 6 | 14 | 3.526 | 30 | -20 | 65 |
| R | FFG | 18 | 15 | 3.521 | 20 | -95 | -20 |
|  |  | 19 | 25 | 3.397 | 20 | -80 | -20 |
|  |  | 37 | 22 | 3.234 | 35 | -55 | -20 |

*The permutation analysis of the regional spectral power in the α2 band revealed significant differences between HC and PwMS in 1237 voxels at a p < 0.05. Sources with a cluster size of fewer than 10 voxels were ignored and are not presented here. PwMS showed decreased spectral power in 19 and 11 sources in the left and right hemispheres, respectively.* *For each source, the hemisphere, anatomical area, and Brodmann area to which it belongs are provided, along with the cluster size (represented as the number of activated voxels), the t-value, and MNI coordinates of the source. For the MNI coordinates, the x coordinate indicates whether the peak voxel is in the left or right hemisphere, the y coordinate indicates whether the peak voxel is in the anterior or the posterior part of the brain, and the z coordinate indicates whether the voxel is in the superior or inferior part of the brain. Abbreviations: BA, Brodmann Area; CS, Cluster Size; T, t-values; R, Right; L, Left; ANG, Angular Gyrus; medFG, Medial Frontal Gyrus; ITG, Inferior Temporal Gyrus; SFG, Superior Frontal Gyrus; MTG, Middle Temporal Gyrus; IPL, Inferior Parietal Lobule; FFG, Fusiform Gyrus; SPL, Superior Parietal Lobule; CUN, Cuneus; MOG, Middle Occipital Gyrus; MFG, Middle Frontal Gyrus; PCUN, Precuneus; SOG, Superior Occipital Gyrus; PCG, Precentral Gyrus; LING, Lingual Gyrus; POG, Postcentral Gyrus; IOG, Inferior Occipital Gyrus; STG, Superior Temporal Gyrus; IFG, Inferior Frontal Gyrus; PCL, Paracentral Lobule.*

## **Correlations**

S4. Association of z-transformed α2 spectral power, and SVF with demographics, disease-related information, PROMs, and cognitive performance at p < 0.05 in PwMS.

|  | α2 power (z-score) | | | SVF | | |
| --- | --- | --- | --- | --- | --- | --- |
|  | *β* | *p* | *adj. p* | *β* | *p* | *adj. p* |
| **Demographics** | | | | | | |
| Age | 0.193 | 0.126 | 0.347 | -0.180 | 0.154 | 0.171 |
| Sex^a^ | 0.339 | 0.307 | 0.563 | 0.155 | 0.633 | 0.633 |
| Education | 0.008 | 0.553 | 0.798 | 0.261 | 0.037 | **0.046** |
| **Disease-Related Information** | | | | | | |
| MS type^a^ | 0.136 | 0.634 | 0.798 | -1.247 | 0.002 | **0.003** |
| EDSS | 0.144 | 0.263 | 0.563 | -0.386 | 0.002 | **0.003** |
| Disease Duration | 0.032 | 0.798 | 0.798 | -0.379 | 0.002 | **0.003** |
| **PROMs** | | | | | | |
| MFIS | 0.225 | 0.074 | 0.271 | -0.393 | 0.001 | **0.003** |
| DASS-21ds | 0.042 | 0.739 | 0.798 | -0.368 | 0.003 | **0.004** |
| MSIS-29 | 0.210 | 0.060 | 0.271 | -0.379 | 0.002 | **0.003** |
| **Cognitive performance**^b^ | | | | | | |
| PVF (XSA) | -0.038 | 0.783 | 0.798 | 0.671 | <0.001 | **0.002** |
| SVF (AFO) | 0.376 | <0.001 | **0.007** | — | — | — |

*CRIq associations with demographics, disease-related information, PROMs, and spectral power are reported in Table 2 of the main manuscript. Here, CRIq associations are reported only for cognitive performance measures. Bold type denotes significance. ^a^ indicates that the logistic regression was applied. ^b^ indicates regression models adjusted for age, sex, MS type, disease duration, MFIS, and DASS-21ds. Abbreviations: SVF, Semantic Verbal Fluency; adj, adjusted; EDSS, Expanded Disability Status Scale; PROMs, Patient/Participant-Reported Outcome Measures; MFIS, Modified Fatigue Impact Scale; DASS-21ds, Depression, Anxiety, Stress Scale – 21 Items depression subscale; MSIS-29, Multiple Sclerosis Impact Scale – 29 Items; PVF, Phonemic Verbal Fluency; XSA, Chi-Sigma-Alpha Phonemic Fluency Test; AFO, Animals-Fruit-Objects Semantic Fluency Test; GVLT, Greek Verbal Learning Test; BVMT-R, Revised Brief Verbal Memory Test; SDMT-Of, Symbol Digit Modalities Test – Oral forma; DS Total, Digit Span Total; SNST, Stroop Neuropsychological Screening Test.*

## **Mediation analysis models**

S5. Model coefficients of the mediation analysis (model 4), with CRIq as an estimator, global α2 spectral power as a mediator, PwMS performance on semantic verbal fluency as an outcome, and age, sex, MS type, disease duration, MFIS, and DASS-21ds as covariates.

|  |  | **Consequent** | | | | | | |
| --- | --- | --- | --- | --- | --- | --- | --- | --- |
|  |  | M (α2) | | |  | Y (AFO/SVF) | | |
| **Antecedent** |  | *Coeff.* | *SE* | *p* |  | *Coeff.* | *SE* | *p* |
| X (CRIq) | *α* | 0.739 | 0.190 | **< 0.001** | *c΄* | 0.251 | 0.159 | 0.120 |
| M (α2) |  | —— | —— | —— | *b* | 0.251 | 0.080 | **0.003** |
| C_1_ (Age) | *f_1_* | 0.017 | 0.013 | 0.204 | *g_1_* | 0.008 | 0.009 | 0.373 |
| C_2_ (Sex) | *f_2_* | -0.073 | 0.381 | 0.849 | *g_2_* | -0.199 | 0.215 | 0.359 |
| C_3_ (MS Type) | *f_3_* | -0.004 | 0.387 | 0.991 | *g_3_* | -0.477 | 0.241 | 0.053 |
| C_4_ (Disease Duration) | *f_4_* | -0.006 | 0.018 | 0.761 | *g_4_* | -0.033 | 0.012 | **0.009** |
| C_5_ (MFIS) | *f_5_* | 0.022 | 0.010 | **0.030** | *g_5_* | -0.011 | 0.008 | 0.160 |
| C_6_ (DASS-21ds) | *f_6_* | -0.023 | 0.039 | 0.549 | *g_6_* | -0.027 | 0.024 | 0.252 |
| Constant | *i_M_* | -1.581 | 0.687 | **0.025** | *i_Y_* | 0.933 | 0.375 | **0.016** |
|  |  | R^2^ = 0.274 | | |  | R^2^ = 0.489 | | |
|  |  | F = 5.730, *p* < **0.001** | | |  | F = 6.803, *p* < **0.001** | | |

*The models presented correspond to the association between CRIq and global α2 spectral power, as well as to the direct effect (regression of CRIq on semantic verbal fluency (AFO), while controlling for α2 spectral power) of CRIq on semantic verbal fluency. The bold type denotes significance at 95% CI. Abbreviations: AFO, Animals-Fruit-Objects Test; SVF, Semantic Verbal Fluency; CRIq, Cognitive Reserve Index questionnaire; Coeff., Coefficient; SE, Standard Error; MFIS, Modified Fatigue Impact Scale; DASS-21ds, Depression, Anxiety, Stress Scale – 21 Items – depression subscale; CI, Confidence Interval.*

S6. Model coefficients of the mediation analysis (model 4), with CRIq-Edu as an estimator, global α2 spectral power as a mediator, PwMS performance on semantic verbal fluency as an outcome, and age, sex, MS type, disease duration, MFIS, and DASS-21ds as covariates.

|  |  | **Consequent** | | | | | | |
| --- | --- | --- | --- | --- | --- | --- | --- | --- |
|  |  | M (α2) | | |  | Y (AFO/SVF) | | |
| **Antecedent** |  | *Coeff.* | *SE* | *p* |  | *Coeff.* | *SE* | *p* |
| X (CRI-Edu) | *α* | 0.208 | 0.213 | 0.334 | *c΄* | 0.216 | 0.137 | 0.122 |
| M (α2) |  | —— | —— | —— | *b* | 0.291 | 0.080 | **0.001** |
| C_1_ (Age) | *f_1_* | 0.016 | 0.014 | 0.269 | *g_1_* | 0.004 | 0.010 | 0.437 |
| C_2_ (Sex) | *f_2_* | 0.455 | 0.452 | 0.318 | *g_2_* | -0.032 | 0.186 | 0.913 |
| C_3_ (MS Type) | *f_3_* | -0.088 | 0.451 | 0.846 | *g_3_* | -0.456 | 0.245 | 0.068 |
| C_4_ (Disease Duration) | *f_4_* | -0.015 | 0.018 | 0.429 | *g_4_* | -0.037 | 0.012 | **0.004** |
| C_5_ (MFIS) | *f_5_* | 0.021 | 0.011 | **0.048** | *g_5_* | -0.01 | 0.008 | 0.194 |
| C_6_ (DASS-21ds) | *f_6_* | -0.043 | 0.039 | 0.278 | *g_6_* | -0.037 | 0.023 | 0.110 |
| Constant | *i_M_* | -1.983 | 0.759 | **0.011** | *i_Y_* | -0.659 | 0.552 | 0.238 |
|  |  | R^2^ = 0.140 | | |  | R^2^ = 0.461 | | |
|  |  | F = 1.298, *p* = 0.269 | | |  | F = 6.446, *p* < **0.001** | | |

*The models presented correspond to the association between the CRI-Edu subscale and global α2 spectral power, as well as to the direct effect (regression of CRI-Edu on semantic verbal fluency (AFO), while controlling for α2 spectral power) of CRI-Edu on semantic verbal fluency. The bold type denotes significance at 95% CI. Abbreviations: AFO, Animals-Fruit-Objects Test; SVF, Semantic Verbal Fluency; CRI-Edu, Cognitive Reserve Index questionnaire – Education subscale; Coeff., Coefficient; SE, Standard Error; MFIS, Modified Fatigue Impact Scale; DASS-21ds, Depression, Anxiety, Stress Scale – 21 Items – depression subscale; CI, Confidence Interval.*

S7. Model coefficients of the mediation analysis (model 4), with CRIq-Leis as an estimator, global α2 spectral power as a mediator, PwMS performance on semantic verbal fluency as an outcome, and age, sex, MS type, disease duration, MFIS, and DASS-21ds as covariates.

|  |  | **Consequent** | | | | | | |
| --- | --- | --- | --- | --- | --- | --- | --- | --- |
|  |  | M (α2) | | |  | Y (AFO/SVF) | | |
| **Antecedent** |  | *Coeff.* | *SE* | *p* |  | *Coeff.* | *SE* | *p* |
| X (CRI-Leis) | *α* | 0.418 | 0.132 | **0.008** | *c΄* | 0.115 | 0.172 | 0.508 |
| M (α2) |  | —— | —— | —— | *b* | 0.282 | 0.088 | **0.002** |
| C_1_ (Age) | *f_1_* | 0.021 | 0.014 | 0.140 | *g_1_* | 0.009 | 0.010 | 0.354 |
| C_2_ (Sex) | *f_2_* | 0.199 | 0.396 | 0.617 | *g_2_* | -0.104 | 0.207 | 0.616 |
| C_3_ (MS Type) | *f_3_* | -0.016 | 0.409 | 0.969 | *g_3_* | -0.485 | 0.241 | **0.049** |
| C_4_ (Disease Duration) | *f_4_* | -0.011 | 0.018 | 0.528 | *g_4_* | -0.034 | 0.013 | **0.009** |
| C_5_ (MFIS) | *f_5_* | 0.023 | 0.010 | **0.030** | *g_5_* | -0.011 | 0.008 | 0.173 |
| C_6_ (DASS-21ds) | *f_6_* | -0.032 | 0.039 | 0.422 | *g_6_* | -0.030 | 0.024 | 0.215 |
| Constant | *i_M_* | -1.894 | 0.688 | **0.008** | *i_Y_* | 0.879 | 0.381 | **0.025** |
|  |  | R^2^ = 0.214 | | |  | R^2^ = 0.477 | | |
|  |  | F = 2.841, *p* = **0.013** | | |  | F = 5.727, *p* < **0.001** | | |

*The models presented correspond to the direct effect (regression of CRI-Leis on semantic verbal fluency (AFO) while controlling for α2 spectral power) of CRI-Leis on semantic verbal fluency. The bold type denotes significance at 95% CI. Abbreviations: CRI-Leis, Cognitive Reserve Index questionnaire – Leisure Subscale; AFO, Animal-Fruit-Objects Test; SVF, Semantic Verbal Fluency; Coeff., Coefficient; SE, Standard Error; MFIS, Modified Fatigue Impact Scale; DASS-21ds, Depression, Anxiety, Stress Scale – 21 Items – depression subscale; CI, Confidence Interval.*

## **Education-Controlled Mediation**

S8. Summary of the mediation analysis for PwMS regarding the effects of different paths with education as an additional covariate.

| **Effect** | **Path** | **Coeff.** | | **SE** | ***t*** | **95% CI** | | ***p*** |
| --- | --- | --- | --- | --- | --- | --- | --- | --- |
|  |  |  |  |  |  | **Lower** | **Upper** |  |
| Total | CRIq → SVF | *c* | 0.507 | 0.253 | 2.001 | -0.001 | 1.015 | 0.050 |
| Direct | CRIq → SVF | *c΄* | 0.285 | 0.239 | 1.194 | -0.194 | 0.756 | 0.238 |
| Indirect | CRIq → α2 → SFV | *ab* | 0.222 | 0.106 | — | **0.049** | **0.456** | — |

*The bold type denotes significance determined by 95% CIs that exclude 0 (as defined by the bootstrap-derived CI lower and upper levels). Abbreviations: Coeff., Coefficient; SE, Standard Error; CI, Confidence Interval; CRIq, Cognitive Reserve Index questionnaire; SVF, Semantic Verbal Fluency test.*

## **DMT-Controlled Mediation**

S9. Summary of the mediation analysis for PwMS regarding the effects of different paths with DMT as an additional covariate.

| **Effect** | **Path** | **Coeff.** | | **SE** | ***t*** | **95% CI** | | ***p*** |
| --- | --- | --- | --- | --- | --- | --- | --- | --- |
|  |  |  |  |  |  | **Lower** | **Upper** |  |
| Total | CRIq → SVF | *c* | 0.434 | 0.171 | 2.539 | **0.091** | **0.777** | **0.014** |
| Direct | CRIq → SVF | *c΄* | 0.251 | 0.16 | 1.57 | -0.069 | 0.571 | 0.122 |
| Indirect | CRIq → α2 → SFV | *ab* | 0.183 | 0.091 | — | **0.041** | **0.387** | — |

*The bold type denotes significance determined by 95% CIs that exclude 0 (as defined by the bootstrap-derived CI lower and upper levels). Abbreviations: Coeff., Coefficient; SE, Standard Error; CI, Confidence Interval; CRIq, Cognitive Reserve Index questionnaire; SVF, Semantic Verbal Fluency test.*

## **Sex-stratified analysis**

S10. Summary of the mediation analysis for women with MS regarding the effects of different paths.

| **Effect** | **Path** | **Coeff.** | | **SE** | ***t*** | **95% CI** | | ***p*** |
| --- | --- | --- | --- | --- | --- | --- | --- | --- |
|  |  |  |  |  |  | **Lower** | **Upper** |  |
| Total | CRIq → SVF | *c* | 0.457 | 0.193 | 2.365 | **0.068** | **0.846** | **0.022** |
| Direct | CRI → SVF | *c΄* | 0.280 | 0.180 | 1.557 | -0.082 | 0.642 | 0.127 |
| Indirect | CRIq → α2 → SFV | *ab* | 0.177 | 0.104 | — | **0.023** | **0.422** | — |

*Significance is determined by 95% CIs that exclude 0 (as defined by the bootstrap-derived CI lower and upper levels). Abbreviations: Coeff., Coefficient; SE, Standard Error; CI, Confidence Interval; CRIq, Cognitive Reserve Index questionnaire; SVF, Semantic Verbal Fluency test.*

S11. Summary of the mediation analysis for men with MS regarding the effects of different paths.

| **Effect** | **Path** | **Coeff.** | | **SE** | ***t*** | **95% CI** | | ***p*** |
| --- | --- | --- | --- | --- | --- | --- | --- | --- |
|  |  |  |  |  |  | **Lower** | **Upper** |  |
| Total | CRIq → SVF | *c* | 0.665 | 0.551 | 1.206 | -0.752 | 2.082 | 0.282 |
| Direct | CRIq → SVF | *c΄* | 0.327 | 0.832 | 0.393 | -1.982 | 2.636 | 0.714 |
| Indirect | CRIq → α2 → SFV | *ab* | 0.338 | 123.504 | — | -12.977 | 15.203 | — |

*Significance is determined by 95% CIs that exclude 0 (as defined by the bootstrap-derived CI lower and upper levels). Abbreviations: Coeff., Coefficient; SE, Standard Error; CI, ConfidenceInterval; CRIq, Cognitive Reserve Index questionnaire; SVF, Semantic Verbal Fluency test.*

## **HC mediation model**

S12. Summary of the mediation analysis for HC regarding the effects of different paths.

| **Effect** | **Path** | **Coeff.** | | **SE** | ***t*** | **95% CI** | | ***p*** |
| --- | --- | --- | --- | --- | --- | --- | --- | --- |
|  |  |  |  |  |  | **Lower** | **Upper** |  |
| Total | CRIq → SVF | *c* | 0.174 | 0.238 | 0.732 | -0.307 | 0.655 | 0.468 |
| Direct | CRIq → SVF | *c΄* | 0.118 | 0.278 | 0.423 | -0.445 | 0.680 | 0.508 |
| Indirect | CRIq → α2 → SFV | *ab* | 0.057 | 0.104 | — | -0.056 | 0.345 | — |

*Significance is determined by 95% CIs that exclude 0 (as defined by the bootstrap-derived CI lower and upper levels). Abbreviations: Coeff., Coefficient; SE, Standard Error; CI, Confidence Interval; CRIq, Cognitive Reserve Index questionnaire; SVF, Semantic Verbal Fluency test.*

S13. Model coefficients of the mediation analysis (model 4), with CRIq as an estimator, global α2 spectral power as a mediator, HC performance on semantic verbal fluency as an outcome, and age, sex, MFIS, and DASS-21ds as covariates.

|  |  | **Consequent** | | | | | | |
| --- | --- | --- | --- | --- | --- | --- | --- | --- |
|  |  | M (α2) | | |  | Y (AFO/SVF) | | |
| **Antecedent** |  | *Coeff.* | *SE* | *p* |  | *Coeff.* | *SE* | *p* |
| X (CRIq) | *α* | -0.056 | 0.044 | 0.218 | *c΄* | 0.118 | 0.278 | 0.120 |
| M (α2) |  | —— | —— | —— | *b* | -1.015 | 1.232 | 0.415 |
| C_1_ (Age) | *f_1_* | 0.000 | 0.001 | 0.905 | *g_1_* | -0.032 | 0.016 | **0.047** |
| C_2_ (Sex) | *f_2_* | 0.017 | 0.060 | 0.778 | *g_2_* | 0.007 | 0.418 | 0.987 |
| C_3_ (MFIS) | *f_3_* | -0.001 | 0.002 | 0.570 | *g_3_* | -0.009 | 0.015 | 0.571 |
| C_4_ (DASS-21ds) | *f_4_* | 0.000 | 0.007 | 0.980 | *g_4_* | -0.028 | 0.040 | 0.498 |
| Constant | *i_M_* | 0.914 | 0.081 | **<0.001** | *i_Y_* | 3.164 | 1.381 | **0.027** |
|  |  | R^2^ = 0.103 | | |  | R^2^ = 0.177 | | |
|  |  | F = 0.434, *p* = 0.822 | | |  | F = 1.900, *p* = 0.105 | | |

*The models presented correspond to the association between CRIq and global α2 spectral power, as well as the direct effect (regression of CRIq on semantic verbal fluency (AFO) while controlling for α2 spectral power) of CRIq on semantic verbal fluency. The bold type denotes significance at 95% CI. Abbreviations: AFO, Animals-Fruit-Objects Test; SVF, Semantic Verbal Fluency; CRIq, Cognitive Reserve Index questionnaire; Coeff., Coefficient; SE, Standard Error; MFIS, Modified Fatigue Impact Scale; DASS-21ds, Depression, Anxiety, Stress Scale – 21 Items – depression subscale; CI, Confidence Interval.*

## **ROI analysis**

S14. Association of z-transformed α2 spectral power in each ROI with CRIq and SVF in PwMS, with age, sex, MS type, disease duration, DASS-21 ds, and MFIS as covariates at p < 0.05 corrected using FDR.

|  | ROI α2 power (z-score) | CRIq | | | SVF | | |
| --- | --- | --- | --- | --- | --- | --- | --- |
|  |  | *β* | *p* | *adj. p* | *β* | *p* | *adj. p* |
| L | Frontal | 0.424 | 0.003 | **0.006** | 0.377 | < 0.001 | **0.001** |
|  | Central | 0.392 | 0.008 | **0.013** | 0.375 | < 0.001 | **0.001** |
|  | Parietal | 0.333 | 0.020 | **0.025** | 0.193 | 0.089 | 0.089 |
|  | Occipital | 0.422 | 0.003 | **0.006** | 0.296 | 0.008 | **0.011** |
|  | Temporal | 0.326 | 0.023 | **0.025** | 0.201 | 0.078 | 0.086 |
| R | Frontal | 0.428 | 0.002 | **0.006** | 0.435 | < 0.001 | **< 0.001** |
|  | Central | 0.467 | 0.001 | **0.006** | 0.339 | 0.002 | **0.003** |
|  | Parietal | 0.484 | < 0.001 | **0.006** | 0.394 | < 0.001 | **0.001** |
|  | Occipital | 0.323 | 0.0252 | **0.025** | 0.389 | < 0.001 | **0.001** |
|  | Temporal | 0.326 | 0.0213 | **0.025** | 0.218 | 0.058 | 0.072 |

*For the regression models with CRIq, α2 spectral power in the corresponding ROIs is the dependent variable; for the regression models with SVF, it is the independent variable. Bold type denotes significance. Abbreviations: ROI, Region of Interest; CRIq, Cognitive Reserve Index questionnaire; SVF, Semantic Verbal Fluency; adj., adjusted; L, Left hemisphere; R, Right hemisphere.*

*S15. Summary of the direct and indirect paths of mediation models with α2 power in each ROI as a mediator at 99% CI level*.

| **Effect** | **Path** | **Coeff.** | | **SE** | ***t*** | **99% CI** | | ***p*** |
| --- | --- | --- | --- | --- | --- | --- | --- | --- |
|  |  |  |  |  |  | **Lower** | **Upper** |  |
| Direct | CRIq → SVF | *c΄* | 0.259 | 0.158 | 1.633 | -0.164 | 0.681 | 0.108 |
| Indirect | CRIq → α2 FL → SVF | *ab* | 0.178 | 0.90 | — | **0.011** | **0.465** | — |
| Direct | CRIq → SVF | *c΄* | 0.271 | 0.176 | 1.538 | -0.199 | 0.740 | 0.130 |
| Indirect | CRIq→ α2 CL → SVF | *ab* | 0.166 | 0.079 | — | **0.007** | **0.421** | — |
| Direct | CRIq → SVF | *c΄* | 0.314 | 0.165 | 1.898 | -0.127 | 0.755 | 0.063 |
| Indirect | CRIq → α2 OL → SVF | *ab* | 0.123 | 0.660 | — | -0.009 | 0.336 | — |
| Direct | CRIq → SVF | *c΄* | 0.219 | 0.153 | 1.430 | -0.189 | 0.626 | 0.158 |
| Indirect | CRIq → α2 FR → SVF | *ab* | 0.219 | 0.100 | — | **0.020** | **0.516** | — |
| Direct | CRIq → SVF | *c΄* | 0.270 | 0.163 | 1.660 | -0.164 | 0.704 | 0.103 |
| Indirect | CRIq → α2 CR → SVF | *ab* | 0.167 | 0.092 | — | -0.009 | 0.469 | — |
| Direct | CRIq → SVF | *c΄* | 0.221 | 0.161 | 1.372 | -0.208 | 0.650 | 0.176 |
| Indirect | CRIq → α2 PR → SVF | *ab* | 0.216 | 0.099 | — | **0.013** | **0.511** | — |
| Direct | CRIq → SVF | *c΄* | 0.293 | 0.156 | 1.881 | -0.122 | 0.708 | 0.065 |
| Indirect | CRIq → α2 OR → SVF | *ab* | 0.144 | 0.087 | — | -0.005 | 0.431 | — |

*The bold type denotes significance, determined by CIs that exclude 0 (as defined by the bootstrap-derived 99% CI lower and upper levels). Abbreviations: ROI, Region of Interest; Coeff., Coefficient; SE, Standard Error; CI, Confidence Interval; CRIq, Cognitive Reserve Index questionnaire; SVF, Semantic Verbal Fluency; FL, Left Frontal ROI; CL, Left Central ROI; PL, Left Parietal ROI; OL, Left Occipital ROI; TL, Left Temporal ROI; FR, Right Frontal ROI; CR, RightCentral ROI; PR, Right Parietal ROI; OR, Right Occipital ROI; TR, Right Temporal Lobe;*

S16. Model coefficients of the mediation analysis (model 4), with CRIq as an estimator, α2 global spectral power in the left frontal ROI as a mediator, PwMS performance on semantic verbal fluency as an outcome, and age, sex, MS type, disease duration, MFIS, and DASS-21ds as covariates.

|  |  | **Consequent** | | | | | | |
| --- | --- | --- | --- | --- | --- | --- | --- | --- |
|  |  | M (α2) | | |  | Y (AFO/SVF) | | |
| **Antecedent** |  | *Coeff.* | *SE* | *p* |  | *Coeff.* | *SE* | *p* |
| X (CRIq) | *α* | 0.807 | 0.282 | **0.006** | *c΄* | 0.259 | 0.158 | 0.108 |
| M (α2 FL) |  | —— | —— | —— | *b* | 0.221 | 0.059 | **< 0.001** |
| C_1_ (Age) | *f_1_* | 0.017 | 0.015 | 0.256 | *g_1_* | 0.009 | 0.009 | 0.352 |
| C_2_ (Sex) | *f_2_* | 0.064 | 0.426 | 0.882 | *g_2_* | -0.232 | 0.201 | 0.255 |
| C_3_ (MS Type) | *f_3_* | 0.01 | 0.441 | 0.982 | *g_3_* | -0.48 | 0.236 | **0.046** |
| C_4_ (Disease Duration) | *f_4_* | -0.002 | 0.024 | 0.932 | *g_4_* | -0.034 | 0.012 | **0.008** |
| C_5_ (MFIS) | *f_5_* | 0.017 | 0.01 | 0.108 | *g_5_* | -0.009 | 0.007 | 0.195 |
| C_6_ (DASS-21ds) | *f_6_* | -0.032 | 0.041 | 0.440 | *g_6_* | -0.026 | 0.023 | 0.267 |
| Constant | *i_M_* | -1.318 | 0.789 | 0.101 | *i_Y_* | 0.827 | 0.361 | **0.026** |
|  |  | R^2^ = 0.221 | | |  | R^2^ = 0.497 | | |
|  |  | F = 3.663, *p* = **0.003** | | |  | F = 7.385, *p* < **0.001** | | |

*The models presented correspond to the association between CRIq and α2 spectral power in the FL ROI, as well as to the direct effect (regression of CRIq on semantic verbal fluency (AFO), while controlling for α2 FL spectral power) of CRIq on semantic verbal fluency. The bold type denotes significance at 99% CI. Abbreviations: ROI, Region of Interest; AFO, Animals-Fruit-Objects Test; SVF, Semantic Verbal Fluency; CRIq, Cognitive Reserve Index questionnaire; Coeff., Coefficient; SE, Standard Error; FL, Left Frontal ROI; MFIS, Modified Fatigue Impact Scale; DASS-21ds, Depression, Anxiety, Stress Scale – 21 Items – depression subscale.*

S17. Model coefficients of the mediation analysis (model 4), with CRIq as an estimator, α2 global spectral power in the left central ROI as a mediator, PwMS performance on semantic verbal fluency as an outcome, and age, sex, MS type, disease duration, MFIS, and DASS-21ds as covariates.

|  |  | **Consequent** | | | | | | |
| --- | --- | --- | --- | --- | --- | --- | --- | --- |
|  |  | M (α2) | | |  | Y (AFO/SVF) | | |
| **Antecedent** |  | *Coeff.* | *SE* | *p* |  | *Coeff.* | *SE* | *p* |
| X (CRIq) | *α* | 1.220 | 0.61 | 0.051 | *c΄* | 0.271 | 0.176 | 0.130 |
| M (α2 CL) |  | —— | —— | —— | *b* | 0.136 | 0.234 | 0.562 |
| C_1_ (Age) | *f_1_* | 0.021 | 0.019 | 0.276 | *g_1_* | 0.010 | 0.010 | 0.342 |
| C_2_ (Sex) | *f_2_* | -0.731 | 0.633 | 0.253 | *g_2_* | -0.118 | 0.227 | 0.606 |
| C_3_ (MS Type) | *f_3_* | -0.305 | 0.590 | 0.607 | *g_3_* | -0.437 | 0.241 | 0.075 |
| C_4_ (Disease Duration) | *f_4_* | 0.032 | 0.044 | 0.471 | *g_4_* | -0.038 | 0.014 | **0.007** |
| C_5_ (MFIS) | *f_5_* | 0.019 | 0.013 | 0.140 | *g_5_* | -0.008 | 0.009 | 0.378 |
| C_6_ (DASS-21ds) | *f_6_* | -0.034 | 0.047 | 0.465 | *g_6_* | -0.029 | 0.025 | 0.263 |
| Constant | *i_M_* | -0.763 | 1.084 | 0.484 | *i_Y_* | 0.640 | 0.445 | 0.156 |
|  |  | R^2^ = 0.163 | | |  | R^2^ = 0.504 | | |
|  |  | F = 2.191, *p* = 0.049 | | |  | F = 5.253, *p* < **0.001** | | |

*The models presented correspond to the association between CRIq and α2 spectral power in the CL ROI, as well as to the direct effect (regression of CRIq on semantic verbal fluency (AFO), while controlling for α2 CL spectral power) of CRIq on semantic verbal fluency. The bold type denotes significance at 99% CI. Abbreviations: ROI, Region of Interest; AFO, Animals-Fruit-Objects Test; SVF, Semantic Verbal Fluency; CRIq, Cognitive Reserve Index questionnaire; Coeff., Coefficient; SE, Standard Error; CL, Left Central ROI; MFIS, Modified Fatigue Impact Scale; DASS-21ds, Depression, Anxiety, Stress Scale – 21 Items – depression subscale; CI, Confidence Interval.*

S18. Model coefficients of the mediation analysis (model 4), with CRIq as an estimator, α2 global spectral power in the right frontal ROI as a mediator, PwMS performance on semantic verbal fluency as an outcome, and age, sex, MS type, disease duration, MFIS, and DASS-21ds as covariates.

|  |  | **Consequent** | | | | | | |
| --- | --- | --- | --- | --- | --- | --- | --- | --- |
|  |  | M (α2) | | |  | Y (AFO/SVF) | | |
| **Antecedent** |  | *Coeff.* | *SE* | *p* |  | *Coeff.* | *SE* | *p* |
| X (CRIq) | *α* | 0.890 | 0.314 | **0.006** | *c΄* | 0.219 | 0.153 | 0.158 |
| M (α2 FR) |  | —— | —— | —— | *b* | 0.246 | 0.056 | < **0.001** |
| C_1_ (Age) | *f_1_* | 0.023 | 0.016 | 0.170 | *g_1_* | 0.007 | 0.009 | 0.449 |
| C_2_ (Sex) | *f_2_* | 0.012 | 0.456 | 0.978 | *g_2_* | -0.221 | 0.202 | 0.280 |
| C_3_ (MS Type) | *f_3_* | -0.131 | 0.509 | 0.797 | *g_3_* | -0.446 | 0.232 | 0.059 |
| C_4_ (Disease Duration) | *f_4_* | -0.006 | 0.025 | 0.813 | *g_4_* | -0.033 | 0.012 | **0.007** |
| C_5_ (MFIS) | *f_5_* | 0.024 | 0.011 | 0.032 | *g_5_* | -0.011 | 0.007 | 0.103 |
| C_6_ (DASS-21ds) | *f_6_* | -0.049 | 0.041 | 0.241 | *g_6_* | -0.021 | 0.023 | 0.351 |
| Constant | *i_M_* | -1.516 | 0.901 | 0.098 | *i_Y_* | 0.909 | 0.351 | 0.012 |
|  |  | R^2^ = 0.252 | | |  | R^2^ = 0.528 | | |
|  |  | F = 4.738, *p* < **0.001** | | |  | F = 8.772, *p* < **0.001** | | |

*The models presented correspond to the association between CRIq and α2 spectral power in the FR ROI, as well as to the direct effect (regression of CRIq on semantic verbal fluency (AFO), while controlling for α2 FR spectral power) of CRIq on semantic verbal fluency. The bold type denotes significance at 99% CI. Abbreviations: ROI, Region of Interest; AFO, Animals-Fruit-Objects Test; SVF, Semantic Verbal Fluency; CRIq, Cognitive Reserve Index questionnaire; Coeff., Coefficient; SE, Standard Error; FR, Right Frontal ROI; MFIS, Modified Fatigue Impact Scale; DASS-21ds, Depression, Anxiety, Stress Scale – 21 Items – depression subscale; CI, Confidence Interval.*

S19. Model coefficients of the mediation analysis (model 4), with CRIq as an estimator, α2 global spectral power in the right parietal ROI as a mediator, PwMS performance on semantic verbal fluency as an outcome, and age, sex, MS type, disease duration, MFIS, and DASS-21ds as covariates.

|  |  | **Consequent** | | | | | | |
| --- | --- | --- | --- | --- | --- | --- | --- | --- |
|  |  | M (α2) | | |  | Y (AFO/SVF) | | |
| **Antecedent** |  | *Coeff.* | *SE* | *p* |  | *Coeff.* | *SE* | *p* |
| X (CRIq) | *α* | 0.860 | 0.250 | **0.001** | *c΄* | 0.221 | 0.161 | 0.176 |
| M (α2 PR) |  | —— | —— | —— | *b* | 0.252 | 0.068 | < **0.001** |
| C_1_ (Age) | *f_1_* | 0.019 | 0.011 | 0.111 | *g_1_* | 0.008 | 0.009 | 0.402 |
| C_2_ (Sex) | *f_2_* | -0.402 | 0.407 | 0.327 | *g_2_* | -0.116 | 0.228 | 0.612 |
| C_3_ (MS Type) | *f_3_* | -0.03 | 0.396 | 0.941 | *g_3_* | -0.471 | 0.246 | 0.061 |
| C_4_ (Disease Duration) | *f_4_* | 0.007 | 0.020 | 0.721 | *g_4_* | -0.036 | 0.012 | **0.004** |
| C_5_ (MFIS) | *f_5_* | 0.016 | 0.009 | 0.071 | *g_5_* | -0.010 | 0.007 | 0.191 |
| C_6_ (DASS-21ds) | *f_6_* | -0.001 | 0.038 | 0.988 | *g_6_* | -0.033 | 0.024 | 0.178 |
| Constant | *i_M_* | -1.283 | 0.692 | 0.069 | *i_Y_* | 0.859 | 0.370 | 0.024 |
|  |  | R^2^ = 0.258 | | |  | R^2^ = 0.503 | | |
|  |  | F = 5.373, *p* < **0.001** | | |  | F = 7.650, *p* < **0.001** | | |

*The models presented correspond to the association between CRIq and α2 spectral power in the PR ROI, as well as to the direct effect (regression of CRIq on semantic verbal fluency (AFO), while controlling for α2 PR spectral power) of CRIq on semantic verbal fluency. The bold type denotes significance at 99% CI. Abbreviations: ROI, Region of Interest; AFO, Animals-Fruit-Objects Test; SVF, Semantic Verbal Fluency; CRIq, Cognitive Reserve Index questionnaire; Coeff., Coefficient; SE, Standard Error; PR, Right Parietal ROI; MFIS, Modified Fatigue Impact Scale; DASS-21ds, Depression, Anxiety, Stress Scale – 21 Items – depression subscale; CI, Confidence Interval.*

## **Moderated mediation analysis models**

S20. Model coefficients of the moderated mediation analysis (model 59), with CRIq as an estimator, global α2 spectral power as mediator, and PwMS performance on semantic verbal fluency as an outcome, with EDSS as moderators of the mediation a-, b-, and direct paths, and age, sex, MS type, disease duration, MFIS, and DASS-21ds as covariates.

|  |  | **Consequent** | | | | | | |
| --- | --- | --- | --- | --- | --- | --- | --- | --- |
|  |  | **EDSS as moderator** | | | | | | |
|  |  | M (α2) | | |  | Y (AFO/SVF) | | |
| **Antecedent** |  | *Coeff.* | *SE* | *p* |  | *Coeff.* | *SE* | *p* |
| X (CRIq) | *α_1_* | 0.620 | 0.395 | 0.122 | *c_1_΄* | 0.321 | 0.337 | 0.345 |
| W (EDSS) | *a_2_* | -0.010 | 0.176 | 0.953 | *c_2_΄* | -0.019 | 0.085 | 0.824 |
| X*W (CRIq x EDSS) | *a_3_* | 0.038 | 0.106 | 0.719 | *c3΄* | -0.021 | 0.081 | 0.799 |
| M (α2) |  | —— | —— | —— | *b_1_* | 0.224 | 0.178 | 0.214 |
| M*W (α2 x EDSS) |  | —— | —— | —— | *b_2_* | 0.010 | 0.055 | 0.858 |
| C_1_ (Age) | *g_1_* | 0.018 | 0.014 | 0.209 | *g_1_* | 0.009 | 0.011 | 0.427 |
| C_2_ (Sex) | *g_2_* | -0.082 | 0.420 | 0.847 | *g_2_* | -0.203 | 0.220 | 0.361 |
| C_3_ (MS Type) | *g_3_* | 0.008 | 0.532 | 0.988 | *g_3_* | -0.444 | 0.277 | 0.115 |
| C_4_ (Disease Duration) | *g_4_* | -0.005 | 0.022 | 0.835 | *g_4_* | -0.032 | 0.013 | **0.014** |
| C_5_ (MFIS) | *g_5_* | 0.021 | 0.010 | 0.046 | *g_5_* | -0.011 | 0.008 | 0.188 |
| C_6_ (DASS-21ds) | *g_6_* | -0.020 | 0.042 | 0.639 | *g_6_* | -0.027 | 0.024 | 0.273 |
| Constant | *i_Y_* | -1.613 | 0.755 | **0.037** | *i_Y_* | 0.945 | 0.440 | **0.037** |
|  |  | R^2^ = 0.276 | | |  | R^2^ = 0.490 | | |
|  |  | F = 4.559, *p* < **0.001** | | |  | F = 4.716, *p* < **0.001** | | |

*The model presented corresponds to the associations between CRIq and α2 spectral power, and the direct effect of CRIq on SVF, moderated by EDSS. The bold type denotes significance at 95% CI. Abbreviations: CRIq, Cognitive Reserve Index questionnaire; EDSS, Expanded Disability Status Scale; AFO, Animals-Fruit-Objects Test; SVF, Semantic Verbal Fluency; Coeff., Coefficient; SE, Standard Error; MFIS, Modified Fatigue Impact Scale; DASS-21ds, Depression, Anxiety, Stress Scale – 21 Items – depression subscale; CI, Confidence Interval.*

S21. Model coefficients of the moderated mediation analysis (model 59), with CRIq as an estimator, global α2 spectral power as mediator, and PwMS performance on semantic verbal fluency as an outcome, with MSIS-29 Physical subscale as moderators of the mediation a-, b-, and direct paths, and age, sex, MS type, disease duration, MFIS, and DASS-21ds as covariates.

|  |  | **Consequent** | | | | | | |
| --- | --- | --- | --- | --- | --- | --- | --- | --- |
|  |  | **MSIS-29 Physical as moderator** | | | | | | |
|  |  | M (α2) | | |  | Y (AFO/SVF) | | |
| **Antecedent** |  | *Coeff.* | *SE* | *p* |  | *Coeff.* | *SE* | *p* |
| X (CRIq) | *α_1_* | 0.302 | 0.590 | 0.612 | *c_1_΄* | 0.231 | 0.402 | 0.568 |
| W (MSIS-29 Physical) | *a_2_* | 0.001 | 0.019 | 0.964 | *c_2_΄* | -0.013 | 0.012 | 0.298 |
| X*W (CRIq x MSIS-29 Physical) | *a_3_* | 0.011 | 0.013 | 0.422 | *c3΄* | 0.001 | 0.009 | 0.937 |
| M (α2) |  | —— | —— | —— | *b_1_* | 0.230 | 0.215 | 0.289 |
| M*W (α2 x MSIS-29 Physical) |  | —— | —— | —— | *b_2_* | 0.000 | 0.005 | 0.930 |
| C_1_ (Age) | *g_1_* | 0.018 | 0.013 | 0.160 | *g_1_* | 0.011 | 0.009 | 0.226 |
| C_2_ (Sex) | *g_2_* | -0.062 | 0.403 | 0.878 | *g_2_* | -0.225 | 0.212 | 0.293 |
| C_3_ (MS Type) | *g_3_* | 0.067 | 0.475 | 0.888 | *g_3_* | -0.322 | 0.291 | 0.274 |
| C_4_ (Disease Duration) | *g_4_* | -0.006 | 0.019 | 0.747 | *g_4_* | -0.033 | 0.012 | **0.010** |
| C_5_ (MFIS) | *g_5_* | 0.022 | 0.012 | 0.073 | *g_5_* | -0.006 | 0.009 | 0.499 |
| C_6_ (DASS-21ds) | *g_6_* | -0.028 | 0.040 | 0.491 | *g_6_* | -0.028 | 0.025 | 0.279 |
| Constant | *i_Y_* | -1.690 | 0.111 | **0.046** | *i_Y_* | 1.096 | 0.498 | **0.032** |
|  |  | R^2^ = 0.283 | | |  | R^2^ = 0.500 | | |
|  |  | F = 5.070, *p* < **0.001** | | |  | F = 6.482, *p* < **0.001** | | |

*The model presented corresponds to the associations between CRIq and α2 spectral power, and the direct effect of CRIq on SVF, moderated by MSIS-29-Physical. The bold type denotes significance at 95% CI. Abbreviations: CRIq, Cognitive Reserve Index questionnaire; MSIS-29-Physical, Multiple Sclerosis Impact Scale-29 Items-Physical Impact Subscale; AFO, Animals-Fruit-Objects Test; SVF, Semantic Verbal Fluency; Coeff., Coefficient; SE, Standard Error; MFIS, Modified Fatigue Impact Scale; DASS-21ds, Depression, Anxiety, Stress Scale – 21 Items – depression subscale; CI, Confidence Interval.*

S22. Model coefficients of the moderated mediation analysis (model 59), with CRIq as an estimator, global α2 spectral power as mediator, and PwMS performance on semantic verbal fluency as an outcome, with MSIS-29 Psychological subscale as moderators of the mediation a-, b-, and direct paths, and age, sex, MS type, disease duration, MFIS, and DASS-21ds as covariates.

|  |  | **Consequent** | | | | | | |
| --- | --- | --- | --- | --- | --- | --- | --- | --- |
|  |  | **MSIS-29 Psychological as moderator** | | | | | | |
|  |  | M (α2) | | |  | Y (AFO/SVF) | | |
| **Antecedent** |  | *Coeff.* | *SE* | *p* |  | *Coeff.* | *SE* | *p* |
| X (CRIq) | *α_1_* | 0.256 | 0.760 | 0.737 | *c_1_΄* | 0.060 | 0.440 | 0.892 |
| W (MSIS-29 Psychological) | *a_2_* | 0.044 | 0.041 | 0.290 | *c_2_΄* | 0.031 | 0.021 | 0.473 |
| X*W (CRIq x MSIS-29 Psychological) | *a_3_* | 0.027 | 0.035 | 0.439 | *c3΄* | 0.012 | 0.023 | 0.599 |
| M (α2) |  | —— | —— | —— | *b_1_* | 0.170 | 0.235 | 0.473 |
| M*W (α2 x MSIS-29 Psychological) |  | —— | —— | —— | *b_2_* | 0.003 | 0.013 | 0.807 |
| C_1_ (Age) | *g_1_* | 0.018 | 0.013 | 0.189 | *g_1_* | 0.009 | 0.009 | 0.322 |
| C_2_ (Sex) | *g_2_* | -0.201 | 0.500 | 0.690 | *g_2_* | -0.306 | 0.220 | 0.170 |
| C_3_ (MS Type) | *g_3_* | -0.078 | 0.371 | 0.835 | *g_3_* | -0.543 | 0.240 | **0.028** |
| C_4_ (Disease Duration) | *g_4_* | 0.001 | 0.019 | 0.951 | *g_4_* | -0.028 | 0.013 | **0.032** |
| C_5_ (MFIS) | *g_5_* | 0.017 | 0.014 | 0.249 | *g_5_* | -0.014 | 0.009 | 0.116 |
| C_6_ (DASS-21ds) | *g_6_* | -0.035 | 0.044 | 0.643 | *g_6_* | -0.036 | 0.023 | 0.114 |
| Constant | *i_Y_* | -2.106 | 0.965 | **0.033** | *i_Y_* | 0.535 | 0.479 | 0.269 |
|  |  | R^2^ = 0.304 | | |  | R^2^ = 0.509 | | |
|  |  | F = 4.729, *p* = **0.005** | | |  | F = 4.743, *p* < **0.001** | | |

*The model presented corresponds to the associations between CRIq and α2 spectral power, and the direct effect of CRIq on SVF, moderated by MSIS-29-Psychological. The bold type denotes significance at 95% CI. Abbreviations: CRIq, Cognitive Reserve Index questionnaire; MSIS-29-Psychological, Multiple Sclerosis Impact Scale-29 Items-Psychological Impact Subscale; AFO, Animals-Fruit-Objects Test; SVF, Semantic Verbal Fluency; Coeff., Coefficient; SE, Standard Error; MFIS, Modified Fatigue Impact Scale; DASS-21ds, Depression, Anxiety, Stress Scale – 21 Items – depression subscale; CI, Confidence Interval.*

## **References**

Benedict RHB, Amato MP, Boringa J, Brochet B, Foley F, Fredrikson S, et al. Brief International Cognitive Assessment for MS (BICAMS): international standards for validation. BMC Neurol 2012;12. https://doi.org/10.1186/1471-2377-12-55.

Kosmidis MH, Vlahou CH, Panagiotaki P, Kiosseoglou G. The verbal fluency task in the Greek population: Normative data, and clustering and switching strategies. Journal of the International Neuropsychological Society 2004;10:164–72. https://doi.org/10.1017/S1355617704102014.

Lezak MD, Howieson DB, Loring DW, Hannay HJ, Fischer JS. Neuropsychological assessment. 4th ed. Oxford University Press; 2004.

Messinis L, Bakirtzis C, Kosmidis MH, Economou A, Nasios G, Anyfantis E, et al. Symbol Digit Modalities Test: Greek Normative Data for the Oral and Written Version and Discriminative Validity in Patients with Multiple Sclerosis. Archives of Clinical Neuropsychology 2021;36:117–25. https://doi.org/10.1093/ARCLIN/ACAA028.

Trenerry MR, Crosson BA, DeBoe J, Leber WR. Stroop neuropsychological screening test. Psychological Assessment Resources 1989.

Vlahou CH, Kosmidis MH, Dardagani A, Tsotsi S, Giannakou M, Giazkoulidou A, et al. Development of the Greek Verbal Learning Test: Reliability, Construct Validity, and Normative Standards. Archives of Clinical Neuropsychology 2013;28:52–64. https://doi.org/10.1093/ARCLIN/ACS099.

Wechsler D. Wechsler Adult Intelligence Scale–Fourth Edition GR: Adaptation for Greece. Motibo Publishing 2014.

Wechsler D. Wechsler Adult Intelligence Scale-Fourth Edition 2008. https://doi.org/10.1037/T15169-000.

Zalonis I, Christidi F, Bonakis A, Kararizou E, Triantafyllou NI, Paraskevas G, et al. The Stroop Effect in Greek Healthy Population: Normative Data for the Stroop Neuropsychological Screening Test. Archives of Clinical Neuropsychology 2009;24:81–8. https://doi.org/10.1093/ARCLIN/ACP011.
